# Supplementary material for: LINC00892 Is an lncRNA Induced by T Cell Activation and Expressed by Follicular Lymphoma-Resident T Helper Cells
Source: Noncoding RNA. 2022 Jun 1;8(3):40. doi: 10.3390/ncrna8030040 (PMC9228450; doi:10.3390/ncrna8030040)
Supplement: Supplementary file 1 [file ncrna-08-00040-s001.zip › ncrna-1713209-supplementary.pdf]

non-coding RNA

MDPI

Supplementary Figures

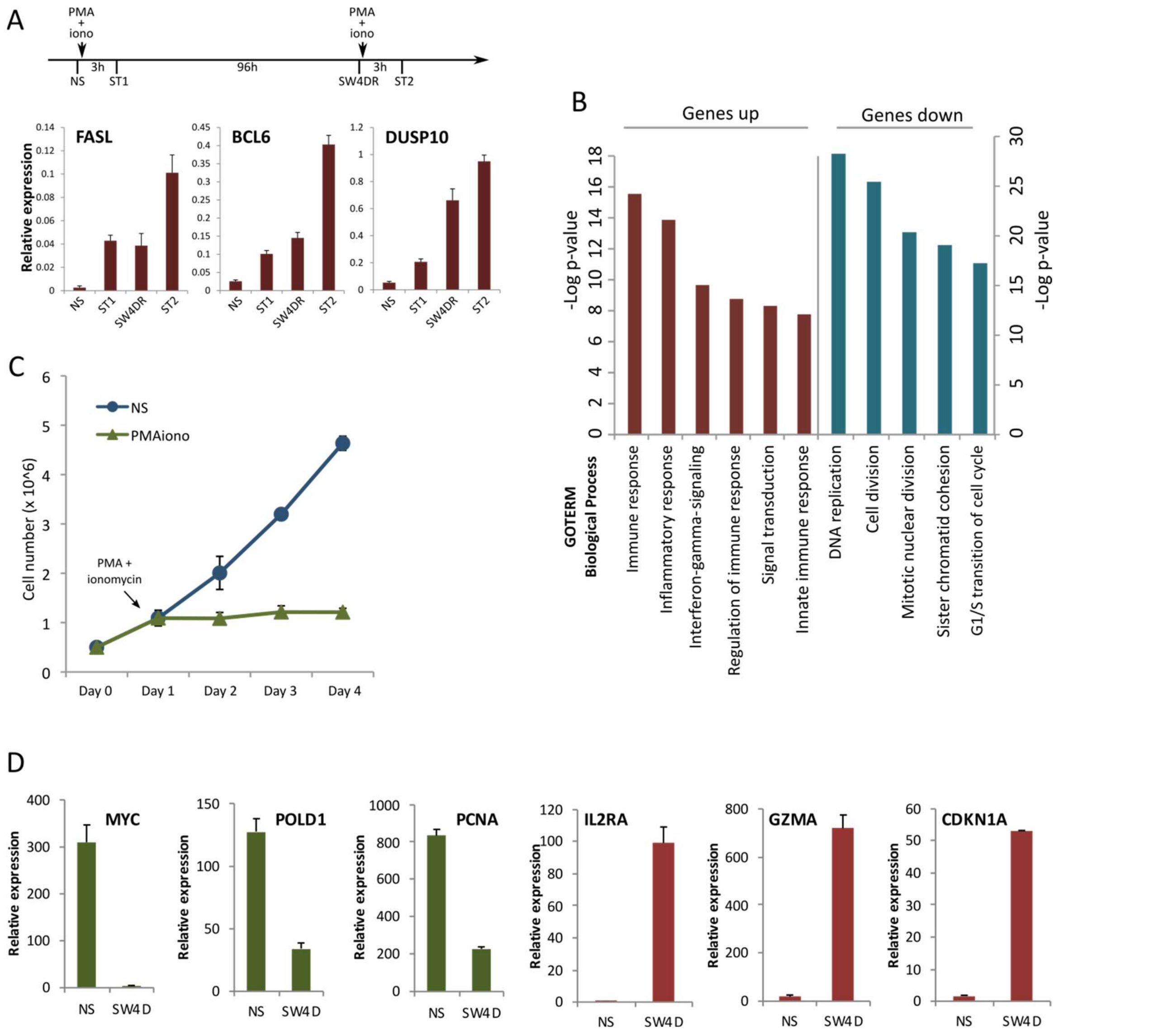

Figure S1. Gene expression analysis in Jurkat cells with a memory-like state, as in [25-27]. (**A**) RT-qPCR analysis of the indicated genes in non-stimulated Jurkat cells (NS), cells stimulated with PMA and ionomycin for 3 hours (ST1), cells stimulated as in ST1 and further grown for 4 days after stimulus withdrawal (SW4D) and SW4D cells stimulated a second time with PMA and ionomycin for 3 hours (ST2). GUSB was used as reference gene. (**B**) Gene ontology analysis of genes differentially expressed in the comparison of NS with SW4D Jurkat cells. The graph shows the significance of the enrichment of the indicated biological processes among both genes up-regulated and down-regulated in SW4D conditions. (**C**) Growth curve of Jurkat cells treated or not treated with a 3 hours pulse of PMA and ionomycin. Analysis has been performed in triplicate. (**D**) Analysis of the expression of the indicated genes in RNA-seq data of Jurkat cells left un-treated or stimulated with PMA/ionomycin for 3 hours ad left untreated for 4 days after stimulus withdrawal (SW4D).

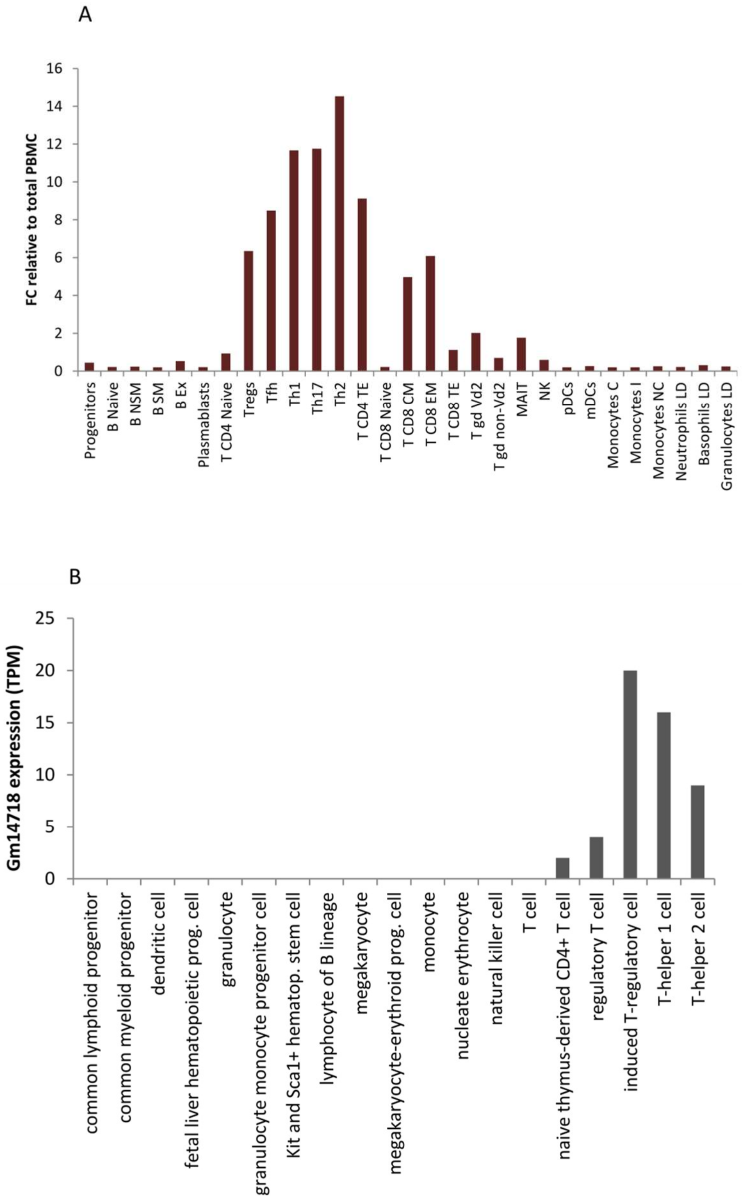

Figure S2. (**A**) Analysis of LINC00892 expression in data from Monaco et al. [30]. (**B**) Analysis of Gm14718 expression in different mononuclear cells derived from peripheral blood (PBMC) in data from the Expression Atlas (https://www.ebi.ac.uk/gxa/home).

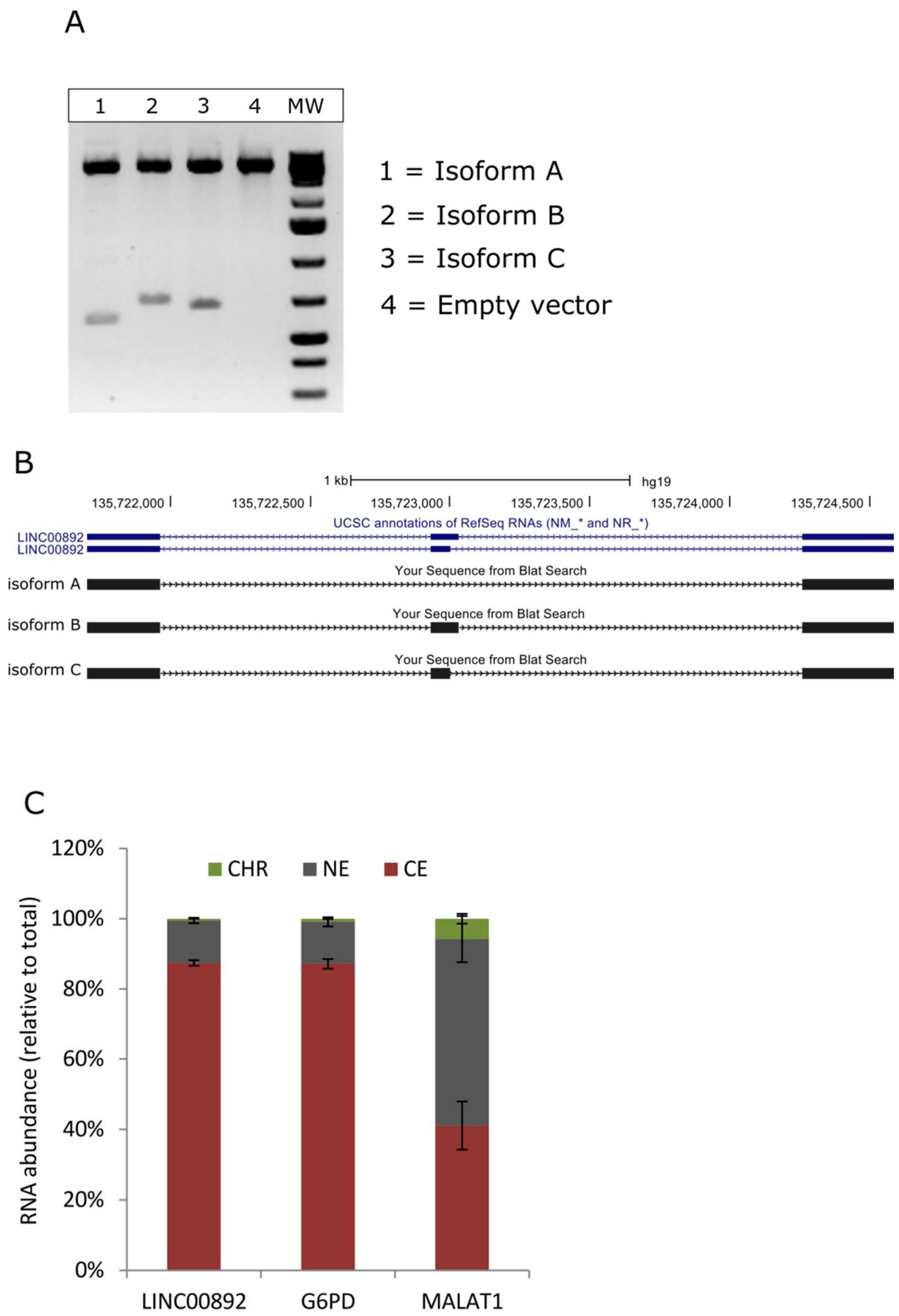

Figure S3. (**A** and **B**) PCR cloning of the LINC00892 cDNA from RNA extracted from a FL patient resulted in the identification of a novel isofom missing exon 2, as depicted in the gene graphic representation shown in panel B. (**C**) RT-qPCR analysis of RNA abundance in cellular fractions from Jurkat cells treated with PMA/ionomycin for 3 hours and collected 48 hours after stimulus wash. CHR is the chromatin fraction; NE is the nuclei enriched fraction and CE is the cytosolic fraction. Expression of G6PD is used as a marker for a typical cytosolic RNA and the expression of MALAT1 is used as a marker for a typical nuclear RNA. The experiment has been performed in quadruplicate.

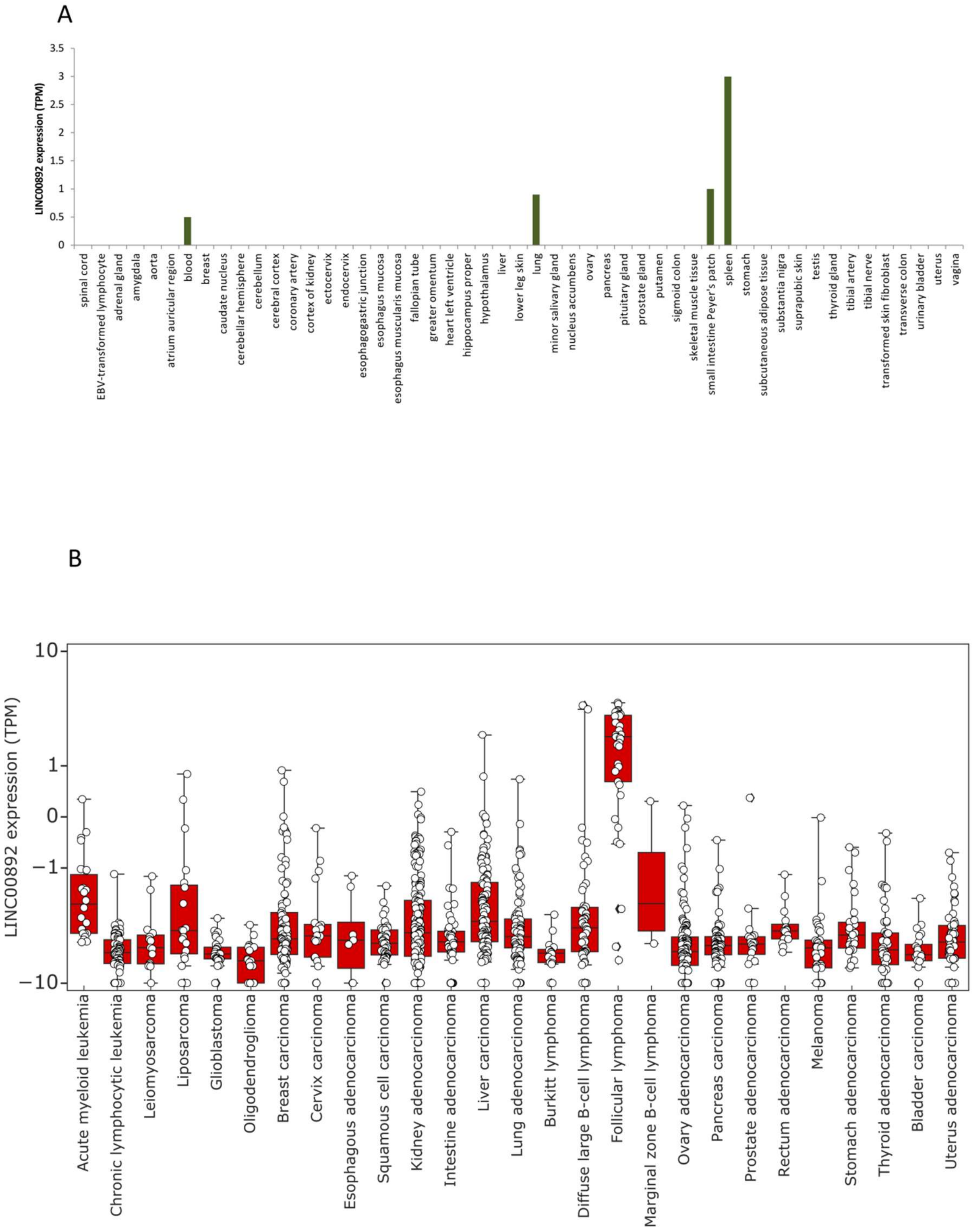

Figure S4. Analysis of LINC00892 expression in normal (**A**) and cancer (**B**) tissues. (**A**) Analysis of LINC00892 expression in data from the Genotype-Tissue Expression (GTEx) Project. (**B**) Analysis of LINC00892 expression in the pan cancer data produced from the International Cancer Genome Consortium (ICGC).

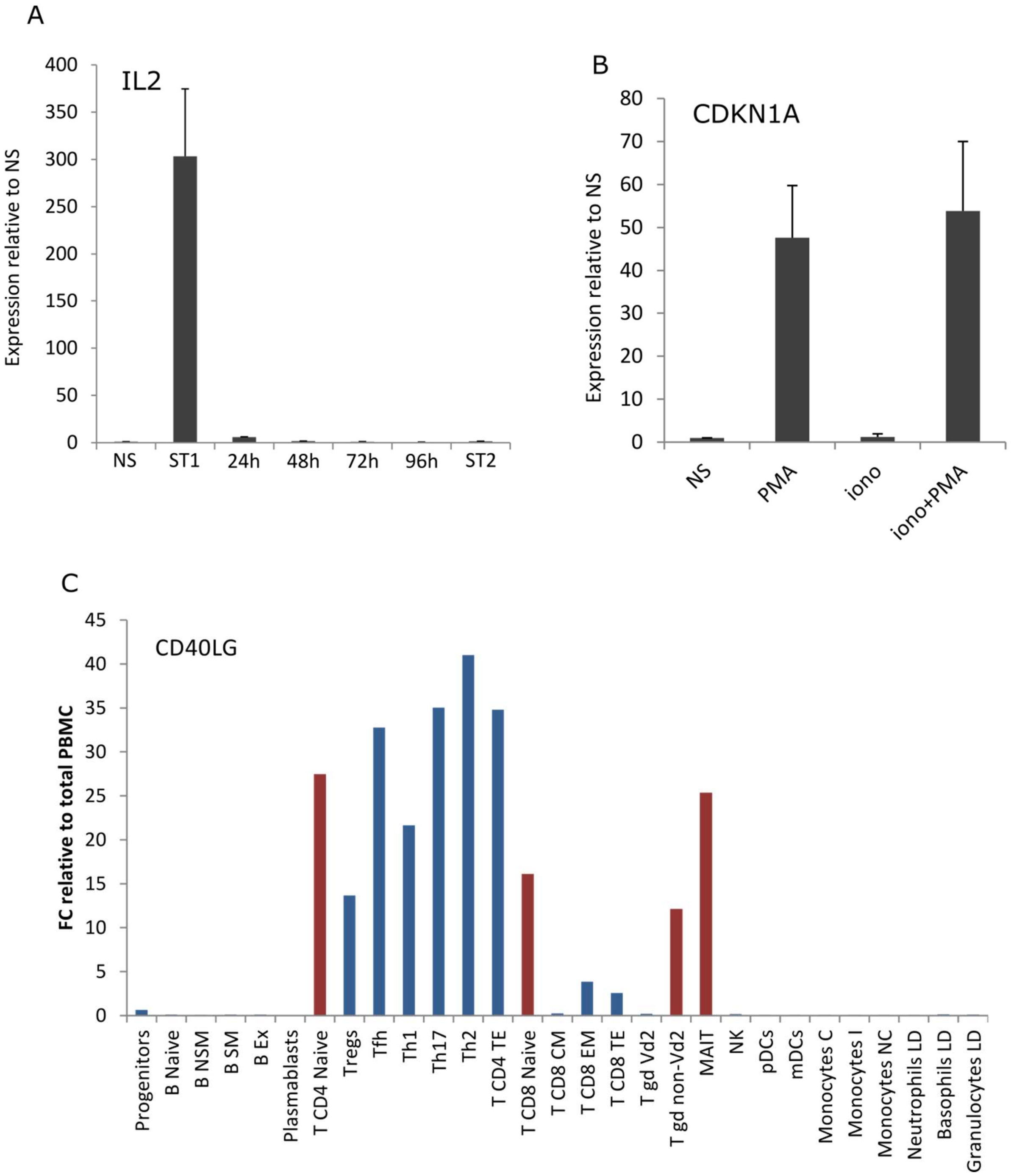

Figure S5. (**A**) RT-qPCR analysis of IL2 expression in Jurkat cells left untreated (NS), stimulated with PMA/ionomycin for 3 hours (ST1), washed and collected after the indicated hours after stimulation, and after a second 3 hours PMA/ionomycin stimulus (ST2). (**B**) RT-qPCR analysis of CDKN1A expression in Jurkat cells left untreated (NS), stimulated with PMA only, ionomycin only or both PMA and ionomycin for 3 hours and collected 4 days after the stimulus. (**C**) Analysis of CD40LG expression in data from Monaco et al. [30]. Cell types where the expression of CD40LG differs from that of LINC00892 are marked in red.

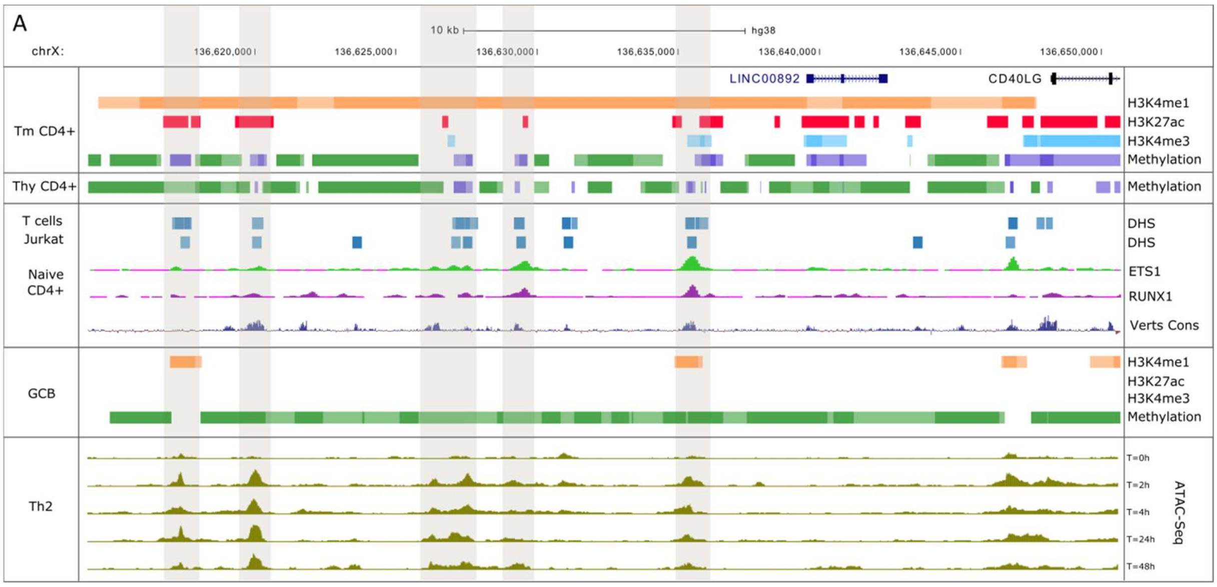

Figure S6. Epigenetic landscape of the genomic locus including LINC00892 and CD40LG. The figure display together publicly available data derived from different sources. On the right side the type of analysis is displayed, on the left the cell lines used for the analyses. Tm CD4+ are CD4+ memory T cells; Thy CD4+ stays for CD4+ thymocytes and GCB for germinal centre-derived B cells. Regions marked by the H3K4me1 histone modification are marked in orange. Regions marked by the H3K27ac histone modification are marked in red. Regions marked by the H3K4me3 histone modification are marked in azure. In the display of methylation data, hypermethylation is shown in green and hypomethylation in purple. ChIP-seq and methylation data are from the Blueprint Project. The figure also shows ATAC-Seq analysis in Th2 cells treated with CD3/CD28 at different time points [31]. (Shown are also the degree of sequence conservation among vertebrates (Verts cons), the presence of DNase hypersensitive sites (DHS) in either Jurkat or CD4+ T cells and the presence of binding sites for the transcription factors ETS1 and RUNX1 in Naive CD4+ T cells [41].

Supplementary Methods

Cloning of LINC00892

In order to be able to express the lncRNA transcript LINC00892 and possibly other lncRNAs, devoid of vector –derived sequences, we modified the mammalian expression vector pcDNA3.1 to remove most of the transcribed regions between the multicloning site and the transcription start site (TSS). The vector was modified by PCR using a forward primer located at the BGH-poly A site having an EcoRI and a BamHI sites in a 5′ tail and a reverse primer located at the TSS with an EcoRI site in a 5′ tail. The PCR product was ligated in order to create the pcDNA_LNC vector having an EcoRI and a BamHI sites between the TSS and the poly-A site. Primer sequences are the following:

BGH-fw :CGGAATTCATAGGATCCTTTCCTATATAAATGAGGAAATTGC

TSS_rev: CGGAATTCTCTCTAGTTAGCCAGAGAGC

In order to clone the LINC00892 transcript in pcDNA_LNC, we performed a reverse transcription reaction from total RNA extracted from an FL sample and used it in a PCR reaction with the following primers:

LINC00892_fw: CGGAATTCAGATGCAGACATGGCTGGATGTTC

LINC00892_rev: CGGGATCCTGATTTATGGGTTATTGTTTATTTTCC

The amplified PCR product has been gel extracted and purified using the Gel extraction Kit from Qiagen, digested with EcoRI and BamHI and cloned in pcDNA_LNC also digested with the same enzymes. Single clonies containing an insert have ben expanded and the purified DNA has been Sanger sequenced.

Nuclear/Cytoplasmic RNA fractionation

A confluent T75 Flask of Jurkat cells is treated with PMA 20ng/ml and ionomycin 500 ng/ml for 3 hours, washed twice with 10 ml full medium and let rest in the incubator for 3 days. Cells are collected by centrifugation and the pellet is washed once with PBS. Washed cells are pelleted, resuspended in 0.5 ml of Buffer A and left on ice for 10 min. After checking under the microscope that more than 90% of the cells are lysed, the cell suspension is centrifuged 10 min at 1000xg at 4 °C to pellet nuclei. The supernatant is transferred to a new tube and is kept as the cytosolic fraction. The nuclear pellet is resuspended in 0.5 ml of Buffer C and left on ice for 10 min. The chromatin fraction is pelleted by centrifugation 10 min at 14000 rpm at 4 °C and the supernatant is transferred in a new tube as nuclear fraction. RNA is extracted from 100 ul of nuclear and cytosolic fractions and from the entire chromatin pellet.

**Buffer A** (hypotonic)	**Buffer C**
Hepes pH 8.0 10 mM	150 mM Nacl
MgCl2 1.5 mM	25 mM Tris pH 8.0
NaCl 10 mM	5 mM EDTA
DTT 0.5 mM	0.5 mM DTT
IGEPAL 0.1%	0.5% IGEPAL
1 x Complete™ (Roche)	1 x Complete™ (Roche)
100 U/ml SUPERase-In (Ambion)	100 U/ml SUPERase-In (Ambion)

Supplementary Tables

Table S1. List of lncRNAs differentially regulated following treatment of Jurkat cells with PMA/ionomicin for 3 hours and collected 4 days after stimulus withdrawal (SW4D).

Geneid	gene_name	logFC	NS 1	NS 2	NS 3	SW4D 1	SW4D 2	SW4D 3	P.Value	adj.P.Val
ENSG00000236039	AC017060.1	9.30	0.00	0.15	0.19	98.40	114.62	105.24	0	0
ENSG00000253138	LINC00967	8.28	0.00	0.08	0.22	49.79	54.19	51.03	7.71E-219	1.78E-216
ENSG00000204054	LINC00963	7.45	0.07	0.05	0.03	10.76	10.46	10.53	1.78E-257	5.46E-255
ENSG00000253490	AC145110.1	7.39	0.00	0.00	0.00	5.34	5.90	5.93	1.25E-102	9.05E-101
ENSG00000258871	RP3-514A23.2	7.38	0.60	0.00	0.00	68.54	73.42	67.74	1.12E-127	1.10E-125
ENSG00000226777	FAM30A	6.97	0.59	0.90	0.72	88.32	81.59	89.20	0	0
ENSG00000233093	LINC00892	6.55	2.55	3.94	3.93	291.30	307.06	310.51	0	0
ENSG00000253522	MIR3142HG	6.54	0.59	1.00	1.40	92.37	84.70	88.37	0.00E+00	7.14E-306
ENSG00000231346	LINC01160	6.42	0.14	0.00	0.31	22.43	20.22	21.50	2.11E-91	1.31E-89
ENSG00000227502	LINC01268	6.30	0.00	0.06	0.00	6.54	7.03	6.12	1.45E-67	6.30E-66
ENSG00000223552	RP11-24F11.2	6.26	0.00	0.50	0.00	27.14	32.29	31.29	6.10E-71	2.79E-69
ENSG00000251191	LINC00589	6.20	0.00	0.00	0.00	6.31	6.81	6.88	2.40E-57	8.62E-56
ENSG00000255201	RP11-350N15.4	5.98	1.46	0.31	4.84	119.29	101.26	106.83	0.0018198	0.0092402
ENSG00000256128	LINC00944	5.98	0.00	0.00	0.00	5.09	7.51	6.76	3.68E-51	1.17E-49
ENSG00000237372	UNQ6494	5.78	0.00	0.00	0.00	3.62	4.48	4.62	4.55E-46	1.30E-44
ENSG00000258623	CTD-2325P2.3	5.72	0.00	0.00	0.00	23.73	20.13	20.63	7.75E-45	2.16E-43
ENSG00000224184	MIR3681HG	5.69	0.00	0.04	0.00	2.38	3.64	3.18	5.94E-49	1.80E-47
ENSG00000227630	LINC01132	5.62	0.00	0.61	0.45	11.31	12.45	11.34	0.0076697	0.0351906
ENSG00000279082	RP1-167O22.1	5.50	0.27	0.15	0.00	6.99	10.55	8.33	5.26E-62	2.08E-60
ENSG00000226088	RP11-180O5.2	5.37	0.68	0.57	1.50	42.90	46.67	48.10	4.74E-75	2.30E-73
ENSG00000271959	CTD-3064M3.7	5.23	0.00	0.00	0.00	3.11	1.91	2.19	2.00E-34	4.40E-33
ENSG00000232895	RP4-543J13.1	5.22	1.18	0.99	0.65	50.39	52.26	55.33	1.77E-57	6.41E-56
ENSG00000227733	RP4-565E6.1	5.19	0.02	0.06	0.06	2.07	1.85	2.53	1.59E-65	6.71E-64
ENSG00000256732	RP11-407A16.3	5.17	0.98	2.07	1.63	53.62	56.70	54.75	3.69E-128	3.66E-126
ENSG00000261504	RP11-317P15.4	5.14	0.00	0.00	0.00	4.05	4.40	5.97	1.02E-32	2.15E-31
ENSG00000246363	RP11-13A1.1	5.14	0.00	0.00	0.00	2.04	2.20	2.42	1.09E-32	2.29E-31
ENSG00000276778	RP11-524N5.1	5.14	0.00	0.00	0.00	1.77	2.05	1.44	1.16E-32	2.44E-31
ENSG00000236230	RP11-400N13.1	5.11	0.00	0.00	0.00	7.94	10.76	9.92	3.28E-32	6.79E-31
ENSG00000251443	RP11-113I22.1	5.06	0.00	0.00	0.00	11.20	11.71	12.96	2.40E-31	4.87E-30
ENSG00000254734	CTD-3138F19.1	5.00	0.34	0.86	1.12	33.14	25.57	36.39	1.65E-56	5.82E-55
ENSG00000258694	RP11-1033H12.1	4.92	2.24	0.00	1.85	44.25	39.89	48.92	7.69E-50	2.37E-48
ENSG00000224950	RP5-1086K13.1	4.78	1.02	0.79	1.22	31.68	22.35	28.35	4.20E-116	3.61E-114
ENSG00000230105	RP11-431N15.2	4.77	0.27	0.46	0.00	10.53	12.73	13.94	1.12E-35	2.54E-34
ENSG00000257242	LINC01619	4.69	0.00	0.05	0.06	2.06	2.38	1.66	7.95E-32	1.63E-30
ENSG00000238178	RP11-431J24.2	4.65	0.00	0.13	0.00	5.28	3.97	4.61	2.33E-27	4.18E-26
ENSG00000261158	CTD-2583P5.3	4.62	0.00	0.00	0.00	2.55	6.75	6.53	1.93E-24	3.15E-23
ENSG00000257023	RP11-268P4.4	4.58	0.23	0.19	0.75	12.94	14.51	10.53	1.64E-38	4.01E-37
ENSG00000238164	RP3-395M20.8	4.55	0.54	0.38	0.67	12.30	9.99	13.31	1.63E-121	1.49E-119
ENSG00000258867	LINC01146	4.54	0.14	0.00	0.23	3.44	3.38	3.77	2.93E-36	6.75E-35
ENSG00000259150	LINC00929	4.51	0.00	0.00	0.00	1.51	3.04	1.95	6.46E-23	1.01E-21

ENSG00000267551	AC005264.2	4.41	0.17	0.21	0.09	6.29	4.09	3.91	5.08E-37	1.20E-35
ENSG00000178977	LINC00324	4.37	1.13	1.90	1.34	27.40	30.43	28.85	6.16E-104	4.51E-102
ENSG00000274021	RP11-823E8.3	4.33	0.00	0.07	0.10	2.00	2.50	3.16	1.44E-25	2.45E-24
ENSG00000227517	LINC01483	4.25	0.00	0.00	0.00	0.82	1.02	0.67	9.22E-20	1.28E-18
ENSG00000248964	RP11-94H18.1	4.23	0.00	0.00	0.00	1.34	1.95	1.67	1.68E-19	2.33E-18
ENSG00000259719	RP11-930O11.1	4.22	0.00	0.00	0.00	2.79	6.68	3.32	2.39E-19	3.29E-18
ENSG00000256995	RP11-114G22.1	4.20	0.00	0.00	0.00	0.79	0.56	0.52	3.65E-19	4.99E-18
ENSG00000274536	MIR223	4.20	8.62	14.58	16.97	238.23	208.66	211.45	3.43E-243	9.76E-241
ENSG00000244620	AL122127.25	4.18	0.81	0.68	0.44	16.80	15.39	20.05	2.06E-30	4.08E-29
ENSG00000245904	RP11-796E2.4	4.11	0.08	0.04	0.09	1.75	1.33	1.37	3.86E-35	8.65E-34
ENSG00000253300	RP11-108E14.1	4.10	0.00	0.00	0.00	2.42	2.80	2.81	5.07E-18	6.62E-17
ENSG00000237311	RP6-159A1.3	4.06	0.00	0.00	0.00	2.50	3.53	4.79	1.30E-17	1.66E-16
ENSG00000238107	RP11-495P10.5	4.05	0.20	0.17	0.11	4.03	3.89	4.06	6.29E-28	1.15E-26
ENSG00000224376	AC017104.6	4.00	0.61	0.10	0.13	5.64	5.43	4.47	1.24E-28	2.32E-27
ENSG00000205890	RP11-473M20.5	3.99	0.13	0.16	0.21	5.04	2.18	3.32	3.42E-32	7.07E-31
ENSG00000237224	RP11-109A6.2	3.99	0.00	0.00	0.09	1.74	2.06	1.01	9.75E-19	1.31E-17
ENSG00000240770	C21orf91-OT1	3.96	0.20	0.33	0.00	5.37	4.45	4.98	1.68E-21	2.51E-20
ENSG00000189238	LINC00943	3.94	0.00	0.00	0.00	0.99	0.52	0.67	2.47E-16	3.01E-15
ENSG00000258987	RP11-131H24.4	3.94	0.00	0.30	0.39	8.92	7.30	7.03	6.11E-20	8.57E-19
ENSG00000271725	RP11-761I4.4	3.91	0.41	0.35	0.45	11.26	13.56	9.08	4.34E-22	6.61E-21
ENSG00000234377	RNF219-AS1	3.90	0.02	0.13	0.05	1.10	0.99	1.13	5.00E-30	9.80E-29
ENSG00000237596	RP13-143G15.4	3.88	0.00	0.02	0.03	0.68	0.67	0.53	2.77E-19	3.80E-18
ENSG00000257495	KRT73-AS1	3.88	0.00	0.32	0.16	2.10	1.77	2.32	1.14E-26	2.00E-25
ENSG00000232679	RP11-400N13.3	3.86	0.00	0.00	0.00	5.34	4.67	3.85	1.46E-15	1.72E-14
ENSG00000226673	LINC01108	3.86	0.05	0.00	0.00	0.81	0.87	0.99	2.89E-17	3.66E-16
ENSG00000227039	ITGB2-AS1	3.85	8.34	15.38	16.60	186.33	153.02	177.80	1.34E-40	3.42E-39
ENSG00000250696	RP11-704M14.1	3.85	0.00	0.00	0.00	0.99	0.60	1.00	1.91E-15	2.24E-14
ENSG00000260641	RP11-1299A16.3	3.81	0.07	0.20	0.27	2.58	2.36	2.56	6.98E-37	1.64E-35
ENSG00000266968	RP11-116O18.1	3.80	0.00	0.00	0.00	0.96	1.24	2.18	5.10E-15	5.89E-14
ENSG00000224413	AP001476.2	3.63	0.00	0.00	0.00	0.98	0.92	1.08	1.53E-13	1.65E-12
ENSG00000276476	LINC00540	3.63	0.00	0.06	0.00	1.10	0.89	0.95	1.32E-14	1.50E-13
ENSG00000272720	CTA-228A9.3	3.62	1.50	1.38	1.65	19.16	19.01	17.49	4.47E-51	1.42E-49
ENSG00000246526	RP11-539L10.2	3.62	0.97	0.90	1.18	13.31	13.19	11.39	1.09E-46	3.17E-45
ENSG00000224228	RP1-15D23.2	3.62	0.00	0.20	0.80	4.78	6.24	5.08	3.54E-18	4.66E-17
ENSG00000226822	RP11-356N1.2	3.61	0.05	0.00	0.00	0.69	0.54	1.05	1.16E-14	1.32E-13
ENSG00000258875	CTD-2547L24.3	3.53	2.25	4.29	3.82	35.82	34.75	38.35	7.28E-90	4.41E-88
ENSG00000226067	LINC00623	3.48	0.47	0.55	0.83	8.06	6.51	6.87	3.14E-34	6.87E-33
ENSG00000250155	CTD-2353F22.1	3.47	0.34	0.28	0.00	4.26	2.44	3.92	1.15E-16	1.42E-15
ENSG00000253177	RP11-100L22.1	3.47	0.00	0.00	0.00	1.48	2.39	1.92	3.24E-12	3.26E-11
ENSG00000227070	RP11-191G24.1	3.44	0.00	0.00	0.00	4.06	5.51	2.72	5.49E-12	5.47E-11
ENSG00000227719	AC006042.6	3.43	0.00	0.00	0.41	6.96	5.39	2.47	4.87E-13	5.13E-12
ENSG00000271992	RP11-42O15.3	3.41	2.52	1.06	0.92	21.17	16.02	15.77	9.20E-24	1.47E-22
ENSG00000229646	RP11-330A16.1	3.40	76.35	45.05	54.57	552.36	579.04	568.36	8.10E-96	5.38E-94
ENSG00000250166	RP11-268P4.5	3.39	0.00	0.38	0.00	5.00	4.70	6.04	1.51E-12	1.55E-11
ENSG00000279110	CTA-243E7.1	3.36	0.03	0.09	0.02	0.36	0.46	0.71	1.67E-20	2.39E-19
ENSG00000204282	TNRC6C-AS1	3.35	3.42	5.79	6.07	46.14	45.47	48.60	3.84E-167	5.60E-165
ENSG00000266709	RP11-214O1.2	3.33	1.42	2.09	1.04	15.71	12.58	15.70	1.30E-43	3.52E-42
ENSG00000260467	RP11-405F3.4	3.32	1.70	1.23	2.41	17.61	17.57	18.55	8.07E-32	1.65E-30
ENSG00000275418	RP11-126O1.6	3.32	0.00	0.00	0.00	5.17	6.48	9.55	4.63E-11	4.39E-10
ENSG00000259881	RP11-830F9.5	3.31	1.54	1.13	0.77	12.56	8.98	10.74	4.58E-52	1.49E-50
ENSG00000267034	RP11-384O8.1	3.31	0.28	0.00	0.15	2.13	2.29	2.45	3.48E-14	3.88E-13
ENSG00000260182	RP11-616M22.5	3.27	0.79	0.00	1.73	12.92	13.78	11.28	7.75E-14	8.48E-13
ENSG00000276691	RP5-1057I20.5	3.26	1.79	1.81	1.58	19.25	17.74	17.41	4.24E-25	7.06E-24

ENSG00000272079	LA16c-380H5.5	3.24	0.15	0.39	0.51	3.38	5.41	3.75	1.56E-17	2.00E-16
ENSG00000273812	WI2-87327B8.2	3.22	0.31	0.26	0.00	3.77	3.55	4.84	2.80E-12	2.83E-11
ENSG00000240535	CTD-2313F11.1	3.21	0.00	0.08	0.10	0.88	0.68	0.84	8.04E-14	8.80E-13
ENSG00000244676	AL109761.5	3.20	0.00	0.00	0.47	5.67	4.00	3.81	3.72E-11	3.54E-10
ENSG00000274020	LINC01138	3.17	0.60	0.79	0.87	6.70	5.74	7.35	7.41E-43	1.97E-41
ENSG00000228863	RP11-404F10.2	3.16	0.05	0.20	0.26	1.51	1.32	1.89	5.54E-19	7.53E-18
ENSG00000274737	RP5-1057I20.6	3.15	0.38	0.32	0.42	7.48	5.08	5.44	5.00E-13	5.25E-12
ENSG00000277851	RP11-756G20.1	3.15	0.73	1.44	0.81	10.99	7.57	10.80	6.24E-21	9.07E-20
ENSG00000267121	CTD-2020K17.1	3.15	6.42	7.47	8.51	63.38	56.75	62.17	2.94E-164	4.18E-162
ENSG00000267414	RP11-456K23.1	3.12	0.19	0.08	0.10	1.66	1.27	2.20	1.29E-13	1.39E-12
ENSG00000265179	RP11-672L10.2	3.11	0.10	0.13	0.17	1.65	0.98	1.66	4.67E-17	5.87E-16
ENSG00000261644	RP11-327F22.1	3.10	0.00	0.14	0.06	0.88	0.77	0.89	2.02E-12	2.05E-11
ENSG00000226330	RP11-739N20.2	3.07	0.00	0.68	0.89	7.94	4.57	6.22	1.87E-12	1.91E-11
ENSG00000235831	BHLHE40-AS1	3.00	1.15	1.88	1.27	10.58	9.88	12.09	3.22E-43	8.65E-42
ENSG00000237346	RP3-448I9.2	3.00	5.20	2.69	3.08	26.32	32.18	27.84	1.03E-27	1.88E-26
ENSG00000277324	RP11-850A17.1	3.00	0.00	0.10	0.00	0.64	1.44	1.03	1.34E-09	1.17E-08
ENSG00000258136	RP11-864J10.4	2.99	0.91	0.48	0.63	5.51	5.77	5.42	4.00E-22	6.08E-21
ENSG00000240527	RP11-429G19.3	2.99	6.33	8.68	9.17	56.27	65.65	59.64	3.08E-48	9.23E-47
ENSG00000266445	RP13-991F5.2	2.98	0.23	0.00	0.26	4.10	1.69	2.58	1.40E-10	1.29E-09
ENSG00000224093	RP5-1033H22.2	2.96	0.00	0.00	0.11	1.21	0.41	1.22	1.79E-09	1.55E-08
ENSG00000237928	NFIA-AS2	2.95	0.64	0.80	0.23	3.61	5.15	4.34	1.10E-19	1.52E-18
ENSG00000260796	RP11-1348G14.5	2.95	0.36	0.00	0.00	3.57	2.24	3.60	2.24E-09	1.92E-08
ENSG00000230400	RP11-359G22.2	2.94	0.18	0.42	0.25	1.83	2.14	2.64	1.61E-21	2.40E-20
ENSG00000278730	RP11-147L13.11	2.93	0.07	0.00	0.07	0.95	0.76	0.52	3.55E-10	3.20E-09
ENSG00000236345	RP11-59D5__B.2	2.91	0.81	0.45	0.44	3.98	5.69	4.46	1.13E-17	1.46E-16
ENSG00000227706	RP11-301G19.1	2.91	1.46	6.04	6.71	29.40	28.28	25.99	0.0101908	0.0456472
ENSG00000270959	LPP-AS2	2.90	0.89	1.27	1.06	7.37	7.99	7.80	1.03E-34	2.27E-33
ENSG00000260273	RP11-425D10.10	2.90	1.12	1.51	0.00	6.39	5.55	4.71	4.32E-14	4.80E-13
ENSG00000240710	RP11-430C7.4	2.89	0.57	0.24	0.63	5.65	4.73	4.43	3.35E-12	3.37E-11
ENSG00000227676	LINC01068	2.87	0.00	0.23	0.00	0.89	4.46	2.09	8.95E-09	7.38E-08
ENSG00000231105	RP5-1071N3.1	2.87	0.08	0.53	0.61	2.77	2.28	2.96	4.62E-17	5.81E-16
ENSG00000223387	RP11-408H1.3	2.86	0.05	0.00	0.12	0.64	0.55	0.71	3.26E-10	2.96E-09
ENSG00000260239	RP11-274H24.1	2.85	0.00	0.43	0.28	1.51	1.42	2.00	1.48E-12	1.52E-11
ENSG00000273174	RP11-434H6.6	2.85	4.15	5.82	4.95	31.51	32.85	40.16	1.52E-31	3.10E-30
ENSG00000226688	ENTPD1-AS1	2.84	1.01	1.54	1.20	7.41	8.71	8.50	3.29E-87	1.92E-85
ENSG00000236751	LINC01186	2.83	0.00	0.00	0.69	2.74	3.87	2.07	4.57E-09	3.84E-08
ENSG00000250274	CTB-114C7.4	2.82	0.09	0.00	0.00	1.08	0.37	0.79	1.44E-08	1.17E-07
ENSG00000242759	LINC00882	2.82	0.76	0.30	0.55	3.70	3.95	3.06	2.77E-25	4.67E-24
ENSG00000258181	RP11-493L12.4	2.82	0.00	0.64	0.84	5.89	5.54	3.39	2.84E-10	2.58E-09
ENSG00000254887	CTC-378H22.1	2.78	0.22	0.55	0.48	3.83	3.37	4.10	1.13E-11	1.11E-10
ENSG00000235492	LINC01221	2.78	4.14	3.23	2.60	23.65	20.42	22.85	7.91E-26	1.35E-24
ENSG00000235304	LINC01281	2.77	0.00	0.19	0.00	0.73	1.02	0.98	1.54E-08	1.25E-07
ENSG00000233791	LINC01136	2.77	0.22	0.42	0.47	2.91	2.81	2.22	3.79E-17	4.77E-16
ENSG00000250739	LINC01262	2.75	0.00	0.00	0.49	0.98	2.98	2.21	1.47E-08	1.19E-07
ENSG00000251301	RP11-81H14.2	2.75	0.22	0.43	0.16	2.15	1.80	1.92	2.39E-14	2.69E-13
ENSG00000253395	KB-1460A1.1	2.73	0.45	0.38	0.50	2.99	7.04	6.53	1.73E-09	1.49E-08
ENSG00000251364	CTD-2516F10.2	2.70	0.72	0.65	0.86	4.53	4.90	4.27	1.67E-40	4.25E-39
ENSG00000232533	AC093673.5	2.69	9.66	8.37	13.29	66.86	57.29	63.69	1.37E-45	3.87E-44
ENSG00000260619	RP11-775C24.3	2.69	0.00	0.47	0.00	0.96	0.74	0.88	4.48E-09	3.77E-08
ENSG00000237914	SIRPG-AS1	2.69	0.07	0.06	0.22	0.87	1.43	0.66	3.23E-10	2.93E-09
ENSG00000226648	PLCG1-AS1	2.68	1.74	1.01	1.77	8.66	10.07	9.31	1.43E-23	2.27E-22
ENSG00000186056	MATN1-AS1	2.68	0.53	0.29	0.54	2.66	2.86	2.95	3.61E-21	5.31E-20
ENSG00000266389	CTB-41I6.1	2.66	1.95	6.14	4.82	22.94	23.09	30.64	9.80E-20	1.36E-18
ENSG00000235890	TSPEAR-AS1	2.66	7.06	6.42	7.22	48.45	35.38	38.72	2.19E-76	1.08E-74

ENSG00000278829	RP11-358B23.7	2.65	1.43	0.80	0.79	8.38	5.91	6.60	2.99E-14	3.35E-13
ENSG00000239636	RP4-728D4.2	2.65	3.42	5.91	7.73	31.03	28.21	37.97	1.65E-39	4.09E-38
ENSG00000262580	RP11-334C17.5	2.64	1.34	1.32	1.60	9.34	6.85	8.86	1.16E-45	3.28E-44
ENSG00000245888	FLJ21408	2.63	0.12	0.35	0.20	1.58	1.36	1.52	5.10E-13	5.36E-12
ENSG00000235586	AC011247.3	2.63	0.00	2.12	0.92	17.49	4.33	8.35	1.07E-08	8.79E-08
ENSG00000248360	LINC00504	2.62	0.00	0.26	0.13	0.42	0.79	0.89	8.03E-11	7.50E-10
ENSG00000226699	RP11-122K13.7	2.61	4.25	1.79	0.00	13.99	9.87	18.79	1.84E-09	1.59E-08
ENSG00000231441	RP11-472M19.2	2.61	0.71	0.00	1.56	5.06	4.76	4.71	1.16E-09	1.01E-08
ENSG00000269843	CTC-490E21.10	2.61	1.62	0.34	0.00	7.12	2.09	6.28	6.30E-09	5.25E-08
ENSG00000232352	SEMA3B-AS1	2.59	0.00	1.46	1.91	5.71	9.55	7.67	2.29E-09	1.96E-08
ENSG00000230454	U73166.2	2.59	0.60	0.50	1.07	4.82	3.54	4.20	9.39E-20	1.31E-18
ENSG00000260409	RP11-403B2.7	2.58	0.49	0.21	0.54	2.28	3.15	2.83	4.54E-12	4.54E-11
ENSG00000266171	RP11-769O8.1	2.55	0.34	0.10	0.38	2.25	1.65	1.77	3.32E-10	3.00E-09
ENSG00000273151	RP11-449P15.2	2.53	1.33	1.00	1.50	7.52	6.45	6.56	1.89E-45	5.32E-44
ENSG00000228382	ITPKB-IT1	2.53	0.67	0.94	1.24	6.90	5.33	5.71	9.15E-13	9.47E-12
ENSG00000261606	RP11-414J4.2	2.52	0.12	0.02	0.00	0.47	0.21	0.29	2.51E-08	2.01E-07
ENSG00000260231	JHDM1D-AS1	2.52	1.31	1.11	0.63	5.68	5.43	5.45	3.37E-21	4.95E-20
ENSG00000236772	RP5-1184F4.5	2.51	0.40	0.33	0.22	3.26	1.64	3.07	5.57E-09	4.66E-08
ENSG00000272030	RP1-178F15.4	2.51	0.19	0.47	1.23	3.66	3.64	3.28	1.01E-10	9.39E-10
ENSG00000280604	PCBP3-OT1	2.51	0.12	0.14	0.00	0.49	0.46	0.67	4.78E-09	4.02E-08
ENSG00000268001	CARD8-AS1	2.51	1.39	2.17	2.73	9.26	12.91	11.19	2.35E-28	4.36E-27
ENSG00000271643	RP11-10C24.3	2.50	0.15	1.43	1.19	6.62	3.99	3.25	4.79E-13	5.05E-12
ENSG00000177406	RP11-218M22.1	2.50	1.05	2.42	2.44	10.39	9.09	9.98	2.54E-37	6.03E-36
ENSG00000245532	NEAT1	2.49	18.41	16.65	21.95	116.30	84.57	93.80	5.59E-05	0.0003399
ENSG00000267737	AC061992.2	2.49	0.00	0.57	0.38	1.87	1.59	2.64	1.68E-08	1.36E-07
ENSG00000244055	AC007566.10	2.48	2.51	0.23	0.92	5.51	6.04	6.47	3.29E-11	3.15E-10
ENSG00000283696	RP11-122G18.12	2.48	1.77	1.65	2.59	10.76	10.73	11.27	1.27E-18	1.70E-17
ENSG00000262319	CTC-457L16.2	2.48	0.07	0.06	0.24	0.80	0.98	0.89	1.35E-08	1.10E-07
ENSG00000281641	SAMD12-AS1	2.47	0.00	0.37	1.22	4.14	2.06	2.46	1.14E-08	9.35E-08
ENSG00000180539	C9orf139	2.46	0.35	0.30	0.30	2.06	1.42	1.91	2.03E-16	2.47E-15
ENSG00000256124	LINC01152	2.46	0.10	0.33	0.05	0.75	0.90	0.97	3.95E-10	3.56E-09
ENSG00000250775	RP11-12K22.1	2.46	0.44	2.20	0.00	4.31	5.40	4.82	3.52E-08	2.79E-07
ENSG00000225431	AP001626.1	2.44	0.55	2.96	2.43	9.30	7.16	9.25	3.35E-16	4.05E-15
ENSG00000264635	RP11-769O8.3	2.44	0.37	0.20	0.13	2.00	2.01	1.08	8.66E-09	7.15E-08
ENSG00000261468	RP11-1024P17.1	2.42	0.51	0.00	0.00	0.78	0.74	1.13	2.92E-07	2.17E-06
ENSG00000237781	RP11-54A4.2	2.40	0.00	0.25	1.30	2.90	2.73	2.92	1.48E-07	1.12E-06
ENSG00000253535	RP11-624C23.1	2.39	0.00	0.37	0.32	1.44	1.50	1.45	1.31E-07	9.94E-07
ENSG00000241155	ARHGAP31-AS1	2.38	0.63	0.00	0.00	1.72	1.62	2.77	1.96E-06	1.36E-05
ENSG00000262001	DLGAP1-AS2	2.37	0.43	0.31	0.41	2.10	1.65	2.52	2.61E-13	2.79E-12
ENSG00000240207	RP11-379F4.4	2.37	0.19	0.39	0.21	1.34	1.45	1.35	2.11E-12	2.14E-11
ENSG00000233355	CHRM3-AS2	2.36	0.45	0.47	0.39	2.48	2.08	2.22	1.78E-15	2.10E-14
ENSG00000162888	C1orf147	2.36	0.28	0.78	1.23	4.40	3.37	2.89	4.66E-14	5.17E-13
ENSG00000270069	MIR222HG	2.36	1.26	0.94	1.62	6.62	6.23	5.66	1.10E-22	1.71E-21
ENSG00000233967	RP11-250B2.3	2.32	0.84	1.23	1.15	4.59	6.91	5.78	1.37E-11	1.34E-10
ENSG00000263466	RP1-56K13.2	2.31	0.45	0.00	0.99	3.44	2.77	4.45	1.22E-06	8.61E-06
ENSG00000248636	RP11-768F21.1	2.31	0.39	0.32	0.00	1.69	2.99	0.85	7.65E-07	5.49E-06
ENSG00000226380	MIR29A	2.30	0.06	0.05	0.16	0.64	0.42	0.39	7.40E-09	6.14E-08
ENSG00000206417	H1FX-AS1	2.30	0.34	0.72	1.01	3.26	2.89	3.10	1.65E-16	2.02E-15
ENSG00000233452	STXBP5-AS1	2.30	0.13	0.08	0.11	0.57	0.37	1.01	2.06E-09	1.77E-08
ENSG00000264548	RP13-516M14.2	2.29	0.08	0.00	0.09	0.72	0.60	0.37	3.32E-06	2.26E-05
ENSG00000266680	RP5-1148A21.3	2.29	0.28	0.35	0.31	1.83	2.30	1.69	3.17E-08	2.52E-07
ENSG00000270604	HCG17	2.29	0.00	0.22	0.28	0.57	2.13	3.13	4.06E-06	2.75E-05
ENSG00000230489	VAV3-AS1	2.28	0.32	0.00	0.70	3.81	3.26	1.05	1.83E-06	1.28E-05
ENSG00000237741	AC002368.4	2.27	0.50	0.42	0.00	1.66	3.12	5.57	5.23E-06	3.50E-05
ENSG00000225339	RP11-513I15.6	2.26	0.07	0.10	0.05	0.67	0.41	0.23	6.39E-09	5.33E-08
ENSG00000268027	AC006129.2	2.26	0.00	0.23	0.15	1.19	0.98	0.75	3.14E-06	2.15E-05

ENSG00000243193	CTA-360L10.1	2.26	0.09	0.00	0.10	0.59	0.46	0.69	4.78E-06	3.22E-05
ENSG00000233695	GAS6-AS1	2.26	1.61	0.83	1.47	6.79	4.90	5.28	1.85E-35	4.18E-34
ENSG00000225335	B476C20.9	2.25	1.66	2.28	2.02	10.36	8.49	8.12	2.94E-23	4.63E-22
ENSG00000260742	RP11-366L5.1	2.25	0.26	0.22	0.17	0.96	1.06	1.36	6.29E-10	5.60E-09
ENSG00000274373	RP13-714J12.1	2.25	0.00	0.23	0.31	3.04	2.29	0.61	6.58E-06	4.37E-05
ENSG00000278869	CITF22-49E9.3	2.24	0.00	0.00	0.98	1.96	1.84	0.99	4.09E-06	2.77E-05
ENSG00000261294	RP11-616M22.3	2.24	1.74	1.95	1.91	10.79	8.95	10.87	6.61E-09	5.50E-08
ENSG00000246898	LINC00920	2.24	2.09	2.83	2.81	12.29	11.46	10.47	5.77E-26	9.91E-25
ENSG00000246145	RRS1-AS1	2.23	0.09	0.19	0.20	0.93	0.65	0.89	5.14E-09	4.31E-08
ENSG00000254419	RP11-261P9.4	2.23	4.75	5.41	7.46	26.39	23.08	28.84	2.28E-19	3.14E-18
ENSG00000262692	CTD-3195I5.3	2.21	6.05	10.17	7.40	39.06	32.59	34.16	1.93E-15	2.26E-14
ENSG00000281961	AF067845.3	2.21	0.81	0.69	1.79	8.04	5.88	4.05	6.15E-08	4.78E-07
ENSG00000245648	RP11-277P12.20	2.21	0.00	0.17	0.08	0.61	0.43	0.53	3.65E-06	2.48E-05
ENSG00000231671	LINC01307	2.21	0.63	1.06	1.39	5.20	3.75	5.41	2.15E-11	2.08E-10
ENSG00000258422	RP11-486O13.2	2.21	0.00	0.15	0.19	1.55	0.91	0.78	1.03E-05	6.74E-05
ENSG00000267138	AC005954.3	2.20	1.16	0.49	0.64	6.36	4.79	4.49	1.56E-06	1.10E-05
ENSG00000255092	RP11-58K22.4	2.20	1.03	0.87	0.38	5.30	3.20	4.57	1.95E-07	1.46E-06
ENSG00000183154	RP11-863K10.7	2.19	0.84	1.58	1.03	5.76	5.61	3.83	2.39E-15	2.80E-14
ENSG00000215769	RP13-104F24.2	2.17	1.74	2.49	2.13	10.73	7.31	9.03	1.50E-26	2.61E-25
ENSG00000272630	RP11-344N10.5	2.16	0.78	1.04	0.64	4.07	3.16	3.45	2.71E-15	3.15E-14
ENSG00000233154	RP4-655J12.4	2.16	0.11	0.10	0.00	0.76	0.47	0.76	1.70E-05	0.0001089
ENSG00000265148	TSPOAP1-AS1	2.16	15.10	16.99	18.42	70.76	63.65	72.49	5.02E-103	3.65E-101
ENSG00000260145	RP11-322D14.2	2.16	0.87	0.73	1.60	7.34	3.60	5.15	1.63E-08	1.32E-07
ENSG00000229401	MIR5689HG	2.15	0.00	0.76	0.00	1.50	1.64	1.01	1.51E-05	9.72E-05
ENSG00000266473	RP11-401F2.3	2.15	0.28	0.24	0.00	2.18	1.46	1.25	1.91E-05	0.0001215
ENSG00000235978	AC018816.3	2.15	2.97	3.55	3.44	14.06	13.39	14.51	3.99E-20	5.65E-19
ENSG00000274184	RP11-158H5.8	2.14	0.19	0.33	0.21	1.17	0.80	2.25	2.16E-07	1.62E-06
ENSG00000234883	MIR155HG	2.13	0.98	1.13	1.21	4.02	6.56	4.32	1.26E-12	1.30E-11
ENSG00000267750	RUNDC3A-AS1	2.13	0.87	1.19	1.24	4.76	4.26	4.44	3.93E-24	6.36E-23
ENSG00000267128	RP11-449J21.5	2.13	0.34	0.29	0.66	2.63	1.86	1.42	2.47E-09	2.11E-08
ENSG00000255299	RP11-655C2.3	2.12	0.98	0.98	1.07	5.57	5.03	3.67	1.12E-09	9.78E-09
ENSG00000245017	RP11-181C3.1	2.12	0.10	0.86	0.45	2.01	1.47	2.03	2.34E-08	1.88E-07
ENSG00000225032	RP11-228B15.4	2.10	3.22	3.44	3.93	14.03	12.39	16.35	6.90E-28	1.26E-26
ENSG00000269243	CTD-2231E14.8	2.10	0.50	0.50	0.55	1.64	2.21	2.92	4.27E-13	4.50E-12
ENSG00000260051	LA16c-390E6.4	2.09	13.04	11.83	12.09	62.46	34.69	54.59	1.63E-25	2.76E-24
ENSG00000215244	RP11-563J2.2	2.09	0.25	0.00	0.21	0.76	0.46	0.77	1.99E-06	1.38E-05
ENSG00000238018	AC093110.3	2.08	2.22	2.53	2.96	12.67	8.65	10.32	3.47E-16	4.20E-15
ENSG00000240990	HOXA11-AS	2.08	0.00	0.11	0.15	0.59	0.42	0.44	1.33E-05	8.60E-05
ENSG00000235609	AF127936.9	2.07	0.13	0.19	0.47	0.87	1.23	0.95	1.46E-10	1.34E-09
ENSG00000250075	RP11-584P21.2	2.07	0.00	0.22	0.09	0.28	0.97	0.66	1.74E-05	0.0001112
ENSG00000261000	RP11-534L20.5	2.07	1.15	0.32	0.42	2.95	3.57	3.82	4.85E-06	3.26E-05
ENSG00000250286	RP11-94C24.8	2.07	0.88	0.74	1.22	5.81	3.42	3.90	2.11E-08	1.70E-07
ENSG00000272168	CASC15	2.07	11.64	12.15	12.77	47.90	45.96	46.91	1.84E-173	2.86E-171
ENSG00000277287	RP4-794I6.4	2.06	0.03	0.09	0.00	0.19	0.21	0.30	1.84E-05	0.0001176
ENSG00000223685	LINC00571	2.06	0.17	0.00	0.18	1.09	0.51	1.28	3.92E-05	0.0002421
ENSG00000263033	RP11-396B14.2	2.05	0.00	0.31	0.20	1.22	0.57	1.84	3.21E-05	0.0001998
ENSG00000271151	RP11-394I13.2	2.05	0.92	0.62	0.81	3.61	3.40	3.44	3.20E-08	2.54E-07
ENSG00000272068	RP11-284F21.9	2.04	2.91	1.63	1.94	8.45	7.51	8.45	8.75E-24	1.40E-22
ENSG00000234817	RP3-400B16.1	2.04	0.69	0.36	0.47	2.07	1.95	2.37	2.54E-09	2.17E-08
ENSG00000225138	CTD-2228K2.7	2.03	16.77	14.52	20.02	73.84	51.21	69.17	1.03E-99	7.20E-98
ENSG00000260740	AC026471.6	2.03	0.00	0.28	0.75	2.23	1.40	2.62	3.69E-05	0.0002288
ENSG00000103472	RRN3P2	2.02	0.42	0.00	0.18	0.73	1.38	0.46	5.61E-06	3.75E-05
ENSG00000256988	RP11-234B24.4	2.02	26.57	43.21	47.48	143.78	140.20	142.94	4.67E-40	1.18E-38
ENSG00000258056	RP11-644F5.11	2.01	2.71	5.01	4.78	16.20	14.82	14.98	4.37E-21	6.41E-20

ENSG00000214894	LINC00243	2.01	0.18	0.23	0.00	0.79	0.56	0.70	1.79E-05	0.0001141
ENSG00000273328	RP11-141M3.6	2.01	0.15	0.04	0.16	0.44	0.56	0.66	2.62E-06	1.80E-05
ENSG00000203804	ADAMTSL4-AS1	2.00	0.22	0.13	0.00	0.65	0.46	0.58	1.93E-05	0.0001231
ENSG00000234261	RP11-146I2.1	2.00	0.11	0.00	0.25	1.61	0.23	0.75	4.41E-05	0.000271
ENSG00000240057	RP11-572M11.4	2.00	1.17	0.99	0.97	3.32	4.23	4.86	7.54E-12	7.46E-11
ENSG00000254815	RP11-496I9.1	1.99	1.21	2.11	2.14	7.01	5.42	7.86	2.44E-17	3.09E-16
ENSG00000224596	ZMIZ1-AS1	1.99	0.35	0.04	0.05	0.39	0.55	0.59	2.64E-06	1.81E-05
ENSG00000260339	HEXA-AS1	1.99	0.44	0.43	0.56	1.84	1.43	2.75	2.58E-09	2.20E-08
ENSG00000237667	LINC01115	1.98	3.35	4.76	4.82	17.93	17.40	12.66	1.18E-21	1.77E-20
ENSG00000247774	PCED1B-AS1	1.98	24.60	27.28	25.07	89.34	94.36	95.89	9.58E-100	6.72E-98
ENSG00000232788	AC078883.3	1.98	0.18	0.76	0.00	0.60	1.50	2.41	2.56E-05	0.0001613
ENSG00000205885	C1RL-AS1	1.98	5.39	4.78	5.72	19.43	19.04	19.11	1.74E-56	6.13E-55
ENSG00000265975	CTB-41I6.2	1.98	3.51	6.20	7.35	20.79	18.84	22.50	4.08E-14	4.54E-13
ENSG00000256967	RP11-273B20.1	1.97	2.49	4.18	6.94	23.29	11.29	14.30	2.41E-18	3.19E-17
ENSG00000254966	RP11-1081L13.4	1.97	1.34	0.56	0.99	5.89	3.23	3.21	1.50E-07	1.13E-06
ENSG00000232043	RP4-530I15.9	1.97	0.09	0.11	0.19	0.37	0.88	0.52	1.33E-06	9.37E-06
ENSG00000259954	IL21R-AS1	1.96	0.07	0.06	0.08	0.66	0.23	0.66	5.00E-05	0.0003056
ENSG00000236151	RP5-1100I6.1	1.95	5.86	2.77	2.82	14.46	13.60	13.35	7.66E-11	7.16E-10
ENSG00000253552	HOXA-AS2	1.95	0.37	0.59	0.24	1.38	1.22	1.80	4.62E-11	4.38E-10
ENSG00000253199	RP11-421P23.1	1.94	0.38	0.16	0.21	1.25	0.78	1.36	3.59E-06	2.44E-05
ENSG00000186594	MIR22HG	1.94	3.08	5.62	6.20	19.18	15.81	15.99	9.82E-33	2.07E-31
ENSG00000254718	CTD-2184C24.2	1.94	0.50	0.57	0.48	2.31	1.54	2.19	6.47E-10	5.76E-09
ENSG00000248740	RP11-328K4.1	1.94	0.21	0.71	0.47	2.79	1.75	1.87	8.99E-06	5.90E-05
ENSG00000239300	RP11-400L8.2	1.93	1.19	0.00	0.44	2.18	2.47	3.08	6.27E-05	0.0003786
ENSG00000272505	RP11-981G7.6	1.92	0.07	0.17	0.23	0.67	0.63	0.76	9.49E-06	6.22E-05
ENSG00000231185	AC005592.2	1.91	0.00	0.18	0.12	0.47	0.67	0.60	0.0001285	0.0007507
ENSG00000267532	MIR497HG	1.90	0.15	0.00	0.11	0.61	0.37	0.22	4.47E-05	0.0002743
ENSG00000254693	RP11-58K22.5	1.90	0.36	0.00	0.79	1.58	2.60	1.99	0.0001165	0.0006849
ENSG00000261366	MANEA-AS1	1.90	0.78	0.65	0.85	3.59	2.05	3.05	9.51E-10	8.38E-09
ENSG00000280303	ERICD	1.90	0.68	1.15	0.75	3.53	2.62	3.34	1.16E-09	1.01E-08
ENSG00000273076	RP3-508I15.22	1.90	1.88	1.98	0.52	4.64	7.28	5.71	3.83E-06	2.60E-05
ENSG00000249502	AC006160.5	1.90	0.78	0.66	0.29	4.00	2.42	1.73	1.28E-05	8.31E-05
ENSG00000233621	LINC01137	1.90	9.11	9.90	4.63	25.68	23.43	29.12	8.45E-26	1.45E-24
ENSG00000225506	CYP4A22-AS1	1.90	0.64	0.71	0.23	2.79	1.75	2.35	8.80E-06	5.78E-05
ENSG00000241525	AC108004.3	1.90	2.51	2.98	2.44	11.83	10.22	6.86	6.57E-14	7.23E-13
ENSG00000274315	RP11-996F15.5	1.89	0.39	1.63	0.00	2.13	2.81	3.01	6.96E-05	0.0004179
ENSG00000260911	RP11-196G11.2	1.89	0.87	0.37	0.48	5.73	3.15	1.44	6.33E-05	0.0003824
ENSG00000244041	LINC01011	1.89	0.83	0.76	1.07	3.05	3.09	3.38	1.75E-11	1.70E-10
ENSG00000230155	RP3-477O4.14	1.88	6.76	6.08	4.36	18.41	19.72	21.12	3.51E-16	4.24E-15
ENSG00000275371	RP11-455F5.6	1.88	1.47	0.46	1.22	5.66	2.66	3.46	1.37E-07	1.03E-06
ENSG00000206028	CTA-373H7.7	1.88	0.22	0.44	0.33	1.07	1.31	1.49	5.56E-07	4.04E-06
ENSG00000262089	RP11-589P10.5	1.88	15.39	14.86	8.79	44.40	38.82	49.77	2.68E-15	3.13E-14
ENSG00000259989	CTD-2555A7.1	1.87	0.41	0.35	0.46	2.27	3.42	1.83	0.0001238	0.0007254
ENSG00000236194	AC003104.1	1.87	0.92	0.88	1.15	5.16	4.18	2.17	9.28E-09	7.64E-08
ENSG00000232677	LINC00665	1.87	0.03	0.05	0.03	0.18	0.28	0.12	8.02E-05	0.0004788
ENSG00000229891	LINC01315	1.87	0.15	1.17	0.00	1.52	1.43	0.85	4.25E-05	0.0002615
ENSG00000272491	RP5-1024N4.4	1.86	0.00	0.20	0.13	0.90	0.60	0.39	0.0001937	0.0011151
ENSG00000223855	HRAT92	1.85	0.78	0.80	1.11	3.67	2.76	2.84	2.22E-12	2.25E-11
ENSG00000257534	RP11-834C11.10	1.84	0.00	0.22	0.57	1.99	1.34	0.86	0.0002203	0.00126
ENSG00000233360	Z83844.1	1.84	0.10	0.17	0.22	1.10	0.62	0.67	6.09E-05	0.0003683

ENSG00000267586	LINC00907	1.84	0.04	0.02	0.02	0.07	0.37	0.09	0.0001032	0.0006101
ENSG00000273096	RP3-508I15.20	1.84	8.55	8.98	5.88	31.64	25.36	27.16	2.55E-08	2.04E-07
ENSG00000259668	RP11-707P17.2	1.84	0.93	1.82	2.73	7.47	5.91	4.62	4.83E-10	4.33E-09
ENSG00000260267	RP11-452L6.5	1.83	11.44	9.45	9.46	34.59	30.20	34.92	7.71E-46	2.19E-44
ENSG00000254614	AP003068.23	1.83	0.66	1.19	0.84	3.35	2.70	3.38	1.08E-08	8.82E-08
ENSG00000232977	LINC00327	1.83	0.24	0.35	0.26	0.99	1.05	1.19	6.35E-07	4.60E-06
ENSG00000274317	RP11-93G5.1	1.83	0.12	0.03	0.13	0.56	0.53	0.17	2.53E-05	0.0001592
ENSG00000229152	ANKRD10-IT1	1.83	16.87	12.13	14.00	61.39	37.58	44.84	4.41E-25	7.35E-24
ENSG00000273443	RP11-54O7.18	1.82	0.71	0.79	2.08	4.40	3.89	3.91	9.66E-07	6.89E-06
ENSG00000258512	LINC00239	1.82	6.43	4.89	3.71	19.13	16.10	14.88	5.06E-12	5.05E-11
ENSG00000267475	CTD-2538C1.2	1.82	2.07	4.34	2.27	9.06	9.24	11.03	1.57E-08	1.28E-07
ENSG00000223979	SMCR2	1.80	0.35	0.29	0.76	2.66	3.58	1.15	0.0001443	0.0008398
ENSG00000245750	DRAIC	1.80	0.14	0.06	0.08	0.46	0.64	0.31	0.0001464	0.0008523
ENSG00000232160	RAP2C-AS1	1.80	0.28	0.19	0.12	0.79	0.68	0.73	6.22E-06	4.14E-05
ENSG00000273901	CTD-2619J13.27	1.80	0.62	1.56	0.34	4.07	1.91	3.76	2.74E-05	0.000172
ENSG00000254092	RP11-369E15.3	1.79	27.20	29.57	29.43	93.61	99.19	84.99	1.08E-24	1.77E-23
ENSG00000260997	RP4-647J21.1	1.78	72.04	90.94	99.72	310.10	253.60	264.15	7.10E-124	6.67E-122
ENSG00000265451	RP11-204L24.2	1.78	0.00	1.69	2.84	2.83	5.03	3.17	2.65E-05	0.0001668
ENSG00000226312	CFLAR-AS1	1.78	0.14	0.04	0.05	0.30	0.37	0.30	0.0001285	0.0007506
ENSG00000281538	RP4-669P10.20	1.78	4.03	5.99	5.80	16.32	17.59	17.47	9.20E-12	9.07E-11
ENSG00000246582	RP11-1149O23.3	1.78	0.32	0.74	0.44	2.02	1.57	1.50	3.67E-07	2.70E-06
ENSG00000261324	RP11-174G6.5	1.76	0.97	0.61	1.07	3.04	3.23	2.15	5.75E-14	6.34E-13
ENSG00000257176	RP11-996F15.2	1.76	1.53	0.99	1.22	3.96	3.51	4.61	6.70E-12	6.66E-11
ENSG00000268069	RP5-1057I20.4	1.76	1.63	0.61	0.80	3.97	2.62	3.60	1.16E-06	8.23E-06
ENSG00000277763	CTD-2588E21.1	1.75	0.58	0.49	0.32	2.54	2.69	1.28	0.000155	0.0009001
ENSG00000227589	RP5-1092A11.5	1.75	0.78	0.65	0.00	1.71	1.61	2.01	0.0001848	0.0010655
ENSG00000215068	AC025171.1	1.75	7.55	9.07	9.24	28.23	24.59	27.35	2.29E-35	5.16E-34
ENSG00000272211	RP11-347P5.1	1.75	0.47	0.24	0.21	1.04	1.07	1.26	1.95E-05	0.000124
ENSG00000272267	RP11-375N15.2	1.75	0.28	0.36	0.31	2.34	1.17	0.78	6.32E-05	0.0003815
ENSG00000231133	HAR1B	1.73	3.06	2.39	1.20	7.42	7.21	6.03	1.96E-08	1.58E-07
ENSG00000223725	AC007879.5	1.73	0.00	0.38	0.50	1.00	1.26	0.84	0.0002184	0.0012493
ENSG00000255355	AP000640.2	1.73	0.21	0.36	0.00	1.41	0.44	1.42	0.0005921	0.0032122
ENSG00000283036	RP11-101C21.4	1.73	0.20	0.33	0.00	1.30	0.41	1.31	0.0005921	0.0032122
ENSG00000205018	RP11-830F9.6	1.73	0.97	1.45	1.43	4.50	3.12	4.89	3.01E-09	2.56E-08
ENSG00000245275	SAP30L-AS1	1.73	0.89	1.17	1.31	3.05	3.68	4.16	5.03E-09	4.22E-08
ENSG00000235852	AC005540.3	1.73	1.06	0.36	0.47	3.03	1.98	1.88	4.07E-05	0.0002509
ENSG00000189223	PAX8-AS1	1.72	0.25	0.46	0.54	1.22	1.27	1.18	2.24E-16	2.73E-15
ENSG00000229771	RP4-644L1.2	1.72	1.05	0.69	0.77	2.83	3.02	2.46	3.20E-07	2.37E-06
ENSG00000233429	HOTAIRM1	1.72	8.50	8.46	10.65	31.40	26.03	26.58	2.74E-24	4.47E-23
ENSG00000267571	AC104532.4	1.71	0.66	1.40	1.10	4.01	3.08	4.40	3.17E-05	0.0001978
ENSG00000231507	LINC01353	1.71	2.34	2.75	6.43	9.22	12.05	11.88	3.34E-10	3.03E-09
ENSG00000255737	AGAP2-AS1	1.70	0.26	0.22	0.86	1.57	1.08	1.73	4.04E-05	0.0002493
ENSG00000182912	TSPEAR-AS2	1.70	4.91	5.16	5.69	18.85	14.34	14.64	4.90E-26	8.44E-25
ENSG00000215067	ALOX12-AS1	1.70	6.81	6.44	4.42	18.73	16.24	17.33	1.43E-28	2.67E-27
ENSG00000260368	RP11-521I2.3	1.69	1.20	0.86	0.00	2.44	1.94	1.14	6.34E-05	0.0003829
ENSG00000247828	TMEM161B-AS1	1.69	6.43	6.07	7.23	20.20	19.09	19.47	4.23E-37	9.97E-36
ENSG00000256576	RP13-977J11.2	1.69	15.55	17.95	19.88	56.80	45.27	57.08	1.06E-34	2.34E-33

ENSG00000232725	U52111.14	1.69	0.54	0.30	0.40	2.57	0.56	2.58	0.0001141	0.0006716
ENSG00000261996	CTC-281F24.1	1.69	19.13	14.78	21.31	59.54	52.11	51.63	1.20E-25	2.04E-24
ENSG00000229056	AC020571.3	1.68	0.97	1.74	1.34	4.00	4.90	3.63	2.03E-08	1.63E-07
ENSG00000249601	LINC01187	1.68	0.17	0.21	0.09	0.65	0.52	0.75	0.0002235	0.0012768
ENSG00000245281	CTD-2547L16.1	1.68	0.53	0.27	0.47	1.64	1.54	1.18	1.85E-05	0.0001179
ENSG00000266947	RP11-799D4.4	1.68	2.43	2.28	3.83	8.77	6.45	10.06	1.13E-16	1.40E-15
ENSG00000271882	KB-1410C5.5	1.67	3.33	2.80	2.62	11.48	5.40	13.67	1.94E-06	1.35E-05
ENSG00000213057	C1orf220	1.66	0.70	0.71	1.09	2.78	2.40	2.41	1.69E-08	1.36E-07
ENSG00000267519	MIR24-2	1.66	1.59	2.14	2.34	6.28	5.69	6.09	8.07E-11	7.54E-10
ENSG00000264968	RP11-387H17.4	1.66	0.81	0.34	0.22	1.56	2.09	1.34	0.000202	0.0011603
ENSG00000275367	RP11-266K4.14	1.66	0.17	0.34	0.26	1.09	0.72	0.77	1.55E-05	9.95E-05
ENSG00000258982	RP11-638I2.4	1.65	4.14	6.46	2.60	14.26	10.36	15.67	1.04E-06	7.40E-06
ENSG00000261971	MMP25-AS1	1.65	6.80	6.36	8.54	24.81	17.70	20.36	3.24E-45	9.07E-44
ENSG00000223745	CCDC18-AS1	1.65	7.15	7.32	8.14	22.50	20.61	22.30	6.88E-40	1.73E-38
ENSG00000226179	LINC00685	1.65	4.57	3.07	2.01	13.03	8.96	8.08	1.48E-06	1.04E-05
ENSG00000267758	RP11-358B23.5	1.65	1.97	0.33	0.43	4.76	2.44	2.18	0.0002797	0.001583
ENSG00000251417	RP11-1348G14.4	1.64	0.40	0.42	0.33	1.75	1.13	1.10	3.13E-05	0.0001955
ENSG00000256312	RP13-977J11.8	1.64	1.00	1.68	5.51	6.58	8.26	7.18	1.75E-05	0.0001121
ENSG00000259514	RP11-685G9.2	1.64	3.61	3.47	3.12	10.19	9.05	11.69	8.55E-09	7.07E-08
ENSG00000225172	RP11-16L9.2	1.64	0.54	0.15	0.00	0.60	0.93	1.20	0.0009582	0.0050506
ENSG00000240137	ERICH6-AS1	1.64	0.95	1.79	0.52	3.63	2.93	3.40	2.28E-05	0.0001439
ENSG00000177337	DLGAP1-AS1	1.64	1.11	1.72	1.74	4.89	3.93	4.62	1.06E-09	9.28E-09
ENSG00000239453	SIDT1-AS1	1.64	5.89	7.43	4.63	12.91	18.66	22.77	5.07E-09	4.25E-08
ENSG00000261596	CTB-31N19.3	1.63	2.13	1.99	1.56	6.48	5.36	5.74	6.23E-07	4.51E-06
ENSG00000281903	AF127936.7	1.63	0.05	0.00	0.12	0.24	0.33	0.18	0.0011466	0.0059835
ENSG00000278156	TSC22D1-AS1	1.63	0.28	0.48	0.56	1.61	0.99	1.44	1.02E-06	7.29E-06
ENSG00000274895	RP11-478J18.2	1.63	1.27	1.06	1.31	3.84	3.38	3.54	1.28E-09	1.12E-08
ENSG00000268204	CTD-3214H19.6	1.62	0.70	0.78	1.02	3.05	3.11	2.31	5.32E-05	0.000324
ENSG00000254531	FLJ20021	1.62	3.96	5.83	5.18	14.39	11.75	17.78	1.25E-10	1.15E-09
ENSG00000228763	LIMS1-AS1	1.62	0.41	0.34	0.45	0.90	3.38	2.27	0.0011251	0.0058796
ENSG00000260136	CTD-2270L9.4	1.62	0.97	1.63	0.86	2.98	3.81	3.86	6.48E-06	4.31E-05
ENSG00000270605	RP5-1092A3.4	1.62	3.02	2.20	3.87	8.38	8.40	8.78	5.41E-13	5.67E-12
ENSG00000235419	AC010149.4	1.61	0.85	0.24	0.62	3.72	1.46	1.56	0.0003949	0.0021913
ENSG00000240143	RP4-753P9.3	1.61	13.20	18.66	15.19	44.74	43.32	46.40	4.55E-12	4.55E-11
ENSG00000262772	RP11-353N14.2	1.61	2.08	3.22	6.98	11.16	10.04	9.91	9.99E-14	1.09E-12
ENSG00000277744	CTC-435M10.12	1.61	0.87	1.83	0.48	5.73	2.70	2.89	0.0002812	0.0015899
ENSG00000153363	LINC00467	1.61	2.29	2.85	3.87	8.25	8.07	8.55	2.07E-20	2.95E-19
ENSG00000223891	OSER1-AS1	1.60	1.34	1.43	1.18	3.63	3.60	4.35	6.92E-09	5.75E-08
ENSG00000214900	LINC01588	1.60	0.33	0.25	0.36	0.92	0.96	0.81	2.71E-11	2.60E-10
ENSG00000277182	CTB-58E17.5	1.60	1.12	0.94	1.23	2.86	3.08	3.81	6.17E-08	4.80E-07
ENSG00000233527	ZNF529-AS1	1.60	4.92	4.22	4.73	12.52	13.39	13.30	5.78E-16	6.92E-15
ENSG00000246339	EXTL3-AS1	1.60	1.33	0.85	1.41	3.32	2.84	3.96	9.73E-12	9.57E-11
ENSG00000258940	RP11-407N17.5	1.60	0.58	0.73	1.11	4.26	1.93	1.75	1.21E-05	7.84E-05
ENSG00000272144	CTD-2035E11.5	1.59	2.24	1.88	1.44	6.96	4.82	4.75	3.27E-07	2.42E-06
ENSG00000261067	RP11-264B17.3	1.58	1.92	1.18	2.03	5.01	4.37	4.56	9.12E-16	1.09E-14
ENSG00000272894	RP5-1159O4.1	1.58	1.24	1.05	1.00	4.09	2.80	2.87	4.72E-07	3.45E-06
ENSG00000229953	RP11-284F21.7	1.58	18.32	15.18	15.06	49.80	41.79	42.76	7.32E-19	9.88E-18
ENSG00000236502	SIX3-AS1	1.57	0.17	0.71	1.87	2.61	1.75	2.25	6.26E-05	0.0003784

ENSG00000271646	RP11-326I11.3	1.57	2.97	2.56	3.44	8.23	8.46	8.12	3.70E-14	4.13E-13
ENSG00000259065	RP5-1021I20.1	1.57	1.14	2.63	0.31	1.87	5.87	3.77	7.87E-05	0.0004699
ENSG00000234084	RP3-388E23.2	1.57	1.36	1.99	1.12	4.83	4.55	4.49	4.87E-05	0.0002978
ENSG00000276633	AJ011931.1	1.57	0.08	0.20	0.09	0.69	0.32	0.43	0.0009609	0.0050645
ENSG00000267270	PARD6G-AS1	1.56	0.17	0.14	0.28	0.61	0.75	0.47	4.71E-05	0.0002885
ENSG00000274565	CTD-3035K23.7	1.56	1.02	0.71	0.56	2.60	2.62	1.87	5.12E-05	0.0003125
ENSG00000259448	RP11-16E12.1	1.55	0.21	0.26	0.79	1.24	1.59	0.80	0.0001274	0.0007447
ENSG00000227579	RP1-35C21.2	1.55	0.00	0.18	0.69	1.37	0.86	0.69	0.0018967	0.0096095
ENSG00000277020	RP11-476H16.1	1.55	0.25	0.84	0.27	1.91	2.31	0.82	0.0009418	0.004973
ENSG00000265474	AC010761.9	1.55	1.50	1.58	1.65	6.59	3.10	5.81	7.25E-05	0.000435
ENSG00000268947	AD000684.2	1.54	3.08	2.10	1.91	6.54	5.76	7.44	1.88E-07	1.41E-06
ENSG00000273061	CDC37L1-AS1	1.54	6.06	4.02	4.57	12.95	10.20	18.33	2.70E-08	2.15E-07
ENSG00000224165	DNAJC27-AS1	1.54	0.97	1.02	0.88	2.39	2.71	2.72	2.70E-11	2.59E-10
ENSG00000255495	AC145124.2	1.54	0.14	0.36	0.16	0.79	0.89	0.79	0.0012795	0.0066326
ENSG00000259536	RP11-111A22.1	1.53	0.42	0.07	0.19	0.46	0.87	0.74	0.0005279	0.0028849
ENSG00000276718	RP1-102E24.10	1.53	3.26	1.65	5.03	8.24	8.08	9.74	1.46E-06	1.02E-05
ENSG00000270820	RP11-355B11.2	1.53	1.00	1.26	1.43	3.50	3.19	3.52	2.19E-07	1.64E-06
ENSG00000267838	AC008746.12	1.53	1.56	1.31	1.96	6.09	4.36	3.68	7.77E-06	5.13E-05
ENSG00000253210	RP11-809O17.1	1.52	2.67	5.09	5.60	13.02	9.25	13.12	9.00E-09	7.42E-08
ENSG00000272941	RP11-134L10.1	1.52	0.85	0.72	0.94	2.25	2.29	3.02	5.86E-05	0.0003555
ENSG00000258949	RP11-857B24.5	1.51	2.72	1.37	1.79	8.94	3.92	6.00	0.0002032	0.0011671
ENSG00000273356	RP11-804H8.6	1.51	0.47	0.64	0.52	1.15	1.96	1.78	3.09E-05	0.0001927
ENSG00000249846	RP11-77P16.4	1.51	0.20	0.25	0.44	0.65	0.82	1.42	0.0003856	0.0021416
ENSG00000180422	LINC00304	1.51	0.89	1.12	0.87	3.47	1.99	2.51	4.37E-09	3.68E-08
ENSG00000277310	RP11-699C17.1	1.51	1.38	1.54	2.02	3.52	6.63	5.58	0.0002033	0.0011677
ENSG00000275294	RP11-428O18.6	1.51	1.25	1.65	1.96	5.47	4.41	3.74	2.73E-06	1.88E-05
ENSG00000251517	RP11-109E24.1	1.50	0.35	0.44	0.00	0.57	1.43	0.77	0.0020768	0.0104646
ENSG00000226239	RP1-310O13.7	1.50	0.10	0.25	0.00	0.32	0.40	0.54	0.0028091	0.0138574
ENSG00000249602	RP11-98D18.3	1.50	7.17	7.75	5.64	16.84	21.13	18.10	1.87E-07	1.40E-06
ENSG00000254028	AC083843.2	1.50	0.23	0.19	0.76	1.01	2.14	1.02	0.0016135	0.008251
ENSG00000235052	RP1-150O5.3	1.50	6.43	10.46	13.22	30.57	25.22	22.27	1.11E-09	9.76E-09
ENSG00000258725	PRC1-AS1	-1.50	1.96	1.28	0.60	0.24	0.34	0.36	0.0003456	0.0019312
ENSG00000254208	RP11-219B4.3	-1.51	2.93	5.27	4.14	0.46	2.16	0.92	0.0004622	0.0025425
ENSG00000225889	AC074289.1	-1.52	0.40	0.94	1.05	0.26	0.08	0.26	0.0004897	0.002686
ENSG00000251396	LINC01301	-1.52	0.40	0.41	0.39	0.19	0.00	0.24	0.0005221	0.002855
ENSG00000258511	RP11-61O1.2	-1.52	3.14	4.84	6.91	0.57	2.16	1.16	0.000496	0.002719
ENSG00000260293	RP11-715J22.6	-1.53	1.32	1.49	1.57	0.46	0.29	0.65	7.79E-08	6.01E-07
ENSG00000247675	LRP4-AS1	-1.53	1.33	1.02	1.20	0.27	0.63	0.13	0.0003824	0.0021251
ENSG00000271803	RP1-63M2.5	-1.53	28.45	20.93	29.85	9.26	5.96	9.33	2.21E-09	1.90E-08

ENSG00000230289	RP11-334J6.6	-1.53	2.07	2.43	3.64	0.91	0.43	0.46	0.0012136	0.0063059
ENSG00000231221	LINC01593	-1.53	9.58	8.48	8.88	1.66	2.08	4.46	2.77E-05	0.0001738
ENSG00000231769	RP1-8B1.4	-1.55	6.09	4.45	6.41	0.58	4.10	1.46	9.71E-06	6.36E-05
ENSG00000269194	AC006942.4	-1.55	2.63	2.53	6.21	1.65	0.00	2.08	0.0003753	0.0020877
ENSG00000272808	RP11-66B24.7	-1.56	0.35	0.55	0.89	0.21	0.12	0.13	6.12E-05	0.0003699
ENSG00000237921	AC004543.2	-1.56	3.18	4.46	4.08	0.58	1.64	0.59	0.0007101	0.0038164
ENSG00000264569	RP13-650J16.1	-1.56	1.93	2.32	2.13	0.30	0.57	0.61	0.0006127	0.0033166
ENSG00000234171	RNASEH1-AS1	-1.57	46.56	38.20	35.53	11.45	13.09	12.24	5.06E-22	7.68E-21
ENSG00000239791	AC002310.7	-1.58	0.61	1.03	0.90	0.67	0.21	0.00	0.0004603	0.0025325
ENSG00000245857	GS1-24F4.2	-1.59	2.05	4.25	4.81	0.90	1.13	0.90	1.63E-06	1.14E-05
ENSG00000267694	RP11-691H4.4	-1.59	12.96	11.89	12.11	4.74	2.03	4.34	3.30E-07	2.44E-06
ENSG00000271936	RP11-443B20.1	-1.60	27.71	30.42	31.09	8.52	8.56	9.56	4.01E-18	5.26E-17
ENSG00000227256	MIS18A-AS1	-1.61	4.32	2.99	3.08	1.39	0.79	0.56	5.49E-05	0.000334
ENSG00000222012	AC005481.5	-1.61	0.91	0.67	0.87	0.31	0.06	0.38	4.04E-05	0.0002493
ENSG00000233922	AL133493.2	-1.61	4.68	4.30	4.62	1.73	1.16	1.21	1.35E-17	1.74E-16
ENSG00000206567	AC022007.5	-1.62	4.09	2.73	4.08	0.93	0.88	1.28	5.61E-09	4.69E-08
ENSG00000239445	ST3GAL6-AS1	-1.62	3.25	3.90	3.58	1.02	0.72	1.03	2.49E-05	0.0001568
ENSG00000272733	KB-208E9.1	-1.63	1.27	1.22	1.20	0.60	0.37	0.00	0.0005677	0.003088
ENSG00000282381	RP11-328P23.4	-1.63	8.50	8.80	7.20	2.87	1.69	2.17	1.16E-06	8.19E-06
ENSG00000179935	LINC00652	-1.64	1.67	2.00	1.79	0.56	0.58	0.39	5.42E-08	4.23E-07
ENSG00000230013	RP11-217B7.3	-1.65	1.61	2.98	1.42	0.35	0.33	0.36	0.000707	0.003801
ENSG00000260423	RP13-735L24.1	-1.65	1.03	1.65	2.01	0.44	0.46	0.34	8.24E-08	6.35E-07
ENSG00000224046	AC005076.5	-1.65	5.73	4.81	3.88	1.45	1.36	0.97	2.60E-06	1.79E-05
ENSG00000245614	DDX11-AS1	-1.65	3.64	3.38	3.00	1.41	0.94	0.59	5.95E-09	4.96E-08
ENSG00000253696	KBTBD11-OT1	-1.65	1.44	2.02	1.85	0.00	1.49	0.27	0.0003854	0.0021408
ENSG00000273162	RP11-108L7.15	-1.67	13.81	11.78	13.88	5.27	2.07	4.42	2.80E-11	2.69E-10
ENSG00000261578	RP11-21L23.2	-1.67	0.37	0.16	0.36	0.00	0.05	0.15	0.0005719	0.0031102
ENSG00000261431	RP4-616B8.4	-1.68	8.37	4.40	4.61	0.00	1.08	2.31	0.0007465	0.0039957
ENSG00000204055	RP11-247A12.2	-1.68	0.34	0.43	0.66	0.19	0.00	0.09	0.000712	0.0038258
ENSG00000269425	AC007292.7	-1.69	9.11	7.66	9.26	2.31	1.45	2.32	3.96E-05	0.0002443
ENSG00000272263	RP11-767C1.2	-1.69	3.45	2.26	5.91	0.42	1.98	0.42	0.0001232	0.0007223
ENSG00000225439	BOLA3-AS1	-1.71	1.60	1.88	1.88	0.35	0.88	0.24	6.95E-06	4.61E-05
ENSG00000233016	SNHG7	-1.71	83.22	78.52	85.68	24.58	21.36	23.62	4.55E-86	2.62E-84
ENSG00000272644	RP11-33O4.1	-1.72	5.58	6.49	9.44	2.82	2.65	0.47	6.56E-06	4.36E-05
ENSG00000231607	DLEU2	-1.73	21.36	25.05	28.97	8.04	6.59	6.18	2.75E-46	7.92E-45
ENSG00000255026	RP11-326C3.2	-1.74	75.72	76.67	100.65	29.43	18.53	21.86	1.05E-32	2.21E-31
ENSG00000254510	RP11-867G23.10	-1.74	21.32	30.91	31.57	7.58	7.43	7.31	7.01E-22	1.06E-20
ENSG00000277342	RP11-843B15.4	-1.75	8.16	8.74	9.81	3.66	3.06	0.82	3.71E-07	2.73E-06
ENSG00000250917	RP4-785G19.5	-1.75	4.09	4.47	3.60	1.79	1.69	0.00	6.44E-05	0.0003886
ENSG00000227036	LINC00511	-1.76	0.52	0.73	0.45	0.25	0.09	0.10	2.56E-07	1.90E-06
ENSG00000262172	CTD-2529O21.1	-1.77	11.04	9.65	11.67	3.87	1.82	2.44	2.53E-07	1.89E-06
ENSG00000248774	RP11-798M19.3	-1.77	6.38	2.21	3.72	0.41	1.16	0.83	4.93E-05	0.0003013
ENSG00000176659	C20orf197	-1.79	19.76	19.08	22.78	5.42	5.47	5.30	2.57E-29	4.92E-28
ENSG00000212719	C17orf51	-1.79	20.20	21.12	20.13	5.63	5.57	5.08	4.22E-60	1.62E-58
ENSG00000260190	RP11-229P13.25	-1.81	1.67	2.21	1.84	0.78	0.00	0.53	9.48E-05	0.0005625
ENSG00000233056	ERVH48-1	-1.81	66.68	72.20	73.72	20.30	18.17	17.18	1.23E-65	5.21E-64

ENSG00000269534	CTC-453G23.5	-1.84	1.94	1.30	2.13	0.64	0.00	0.64	4.54E-05	0.0002785
ENSG00000268555	RP11-678G14.3	-1.84	17.43	21.08	23.76	4.37	6.17	5.14	1.44E-18	1.92E-17
ENSG00000262712	RP11-295D4.1	-1.84	2.11	3.21	2.98	0.44	0.83	0.67	3.67E-08	2.90E-07
ENSG00000257497	RP11-585P4.5	-1.85	4.62	4.88	3.63	0.72	1.09	1.31	4.89E-09	4.11E-08
ENSG00000272942	CTA-246H3.12	-1.85	5.76	1.76	4.61	1.44	0.27	0.87	4.80E-06	3.22E-05
ENSG00000281344	HELLPAR	-1.85	0.04	0.03	0.06	0.01	0.01	0.01	2.39E-10	2.19E-09
ENSG00000226791	AC109826.1	-1.85	23.46	27.70	22.55	5.51	6.43	6.58	2.32E-29	4.45E-28
ENSG00000225964	NRIR	-1.85	1.21	2.37	1.11	0.44	0.00	0.44	8.91E-05	0.0005302
ENSG00000251556	RP11-118M9.3	-1.86	4.90	5.24	5.89	1.47	1.38	0.49	1.07E-05	6.97E-05
ENSG00000260500	CTD-3193O13.1	-1.86	3.48	6.50	5.53	3.81	0.00	1.28	1.07E-05	6.99E-05
ENSG00000237356	AL163953.3	-1.86	5.92	6.43	6.41	1.30	1.41	1.91	1.82E-13	1.95E-12
ENSG00000276384	RP11-186B7.7	-1.88	3.21	4.25	5.56	1.51	1.42	0.00	2.96E-05	0.0001853
ENSG00000222043	AC079305.10	-1.90	2.74	1.98	1.73	0.00	0.00	1.30	0.0001676	0.00097
ENSG00000260260	SNHG19	-1.90	147.53	148.56	173.14	47.66	34.22	33.49	3.30E-31	6.68E-30
ENSG00000281849	RP13-465B17.4	-1.91	2.83	4.42	3.11	0.00	2.08	0.45	3.63E-05	0.0002257
ENSG00000269951	RP11-797A18.6	-1.91	6.98	1.71	6.73	2.55	0.30	0.64	1.85E-06	1.29E-05
ENSG00000257167	TMPO-AS1	-1.91	10.49	16.70	18.79	4.22	3.13	3.54	3.90E-30	7.67E-29
ENSG00000215458	AATBC	-1.92	0.60	0.64	0.66	0.09	0.17	0.13	1.12E-06	7.96E-06
ENSG00000259343	TMC3-AS1	-1.92	4.78	5.48	3.35	0.79	1.05	1.12	3.69E-09	3.12E-08
ENSG00000254703	SENCR	-1.92	5.42	8.11	8.63	1.49	2.02	1.50	1.49E-11	1.45E-10
ENSG00000253174	RP11-360L9.7	-1.92	1.97	0.97	0.91	0.18	0.00	0.55	5.50E-05	0.0003346
ENSG00000228649	AC005682.5	-1.93	11.17	8.95	9.84	2.29	2.23	2.55	9.40E-21	1.36E-19
ENSG00000276571	AC002550.6	-1.93	6.45	3.34	6.56	0.54	2.05	0.55	1.08E-05	7.02E-05
ENSG00000281398	SNHG4	-1.95	77.88	52.76	61.60	16.82	13.62	14.70	9.66E-54	3.23E-52
ENSG00000268240	RP11-678G14.4	-1.96	0.82	0.50	0.16	0.00	0.08	0.08	9.19E-05	0.0005459
ENSG00000163597	SNHG16	-1.97	59.74	64.55	61.64	13.92	14.60	15.11	3.21E-102	2.30E-100
ENSG00000227848	SUCLA2-AS1	-1.97	1.77	3.28	5.46	0.78	0.37	0.39	1.59E-05	0.0001024
ENSG00000268649	MIR296	-1.97	5.27	5.26	3.26	0.72	1.02	0.73	1.68E-06	1.17E-05
ENSG00000276672	RP11-142E9.1	-1.99	4.85	4.89	5.20	1.43	1.18	0.84	3.42E-20	4.84E-19
ENSG00000265933	LINC00668	-2.00	0.16	0.34	0.40	0.04	0.00	0.09	3.69E-05	0.0002292
ENSG00000248015	AC005329.7	-2.01	2.79	3.78	3.80	0.66	0.45	1.33	2.88E-14	3.23E-13
ENSG00000269846	RP4-621N11.2	-2.01	0.98	0.71	1.39	0.15	0.00	0.31	3.15E-05	0.0001965
ENSG00000255717	SNHG1	-2.02	204.15	190.86	216.01	48.77	48.40	41.65	4.34E-180	7.30E-178
ENSG00000227301	RP11-384P7.5	-2.02	1.20	1.29	1.13	0.19	0.35	0.00	2.91E-05	0.0001818
ENSG00000176124	DLEU1	-2.03	4.26	4.11	4.13	1.03	0.92	0.84	1.08E-32	2.27E-31
ENSG00000270988	RP11-439C15.5	-2.03	1.57	3.31	2.60	0.43	0.00	0.43	4.62E-05	0.0002831
ENSG00000271989	RP4-736L20.3	-2.03	1.23	1.44	1.89	0.27	0.00	0.27	4.48E-05	0.0002752
ENSG00000259959	RP11-121C2.2	-2.06	3.66	4.79	4.75	1.02	0.86	0.92	1.37E-18	1.82E-17
ENSG00000272259	RP11-305P22.9	-2.06	1.74	0.71	0.93	0.22	0.15	0.22	2.24E-08	1.80E-07
ENSG00000276390	RP1-197B17.5	-2.07	4.92	3.82	5.83	0.83	1.17	0.42	1.01E-06	7.22E-06
ENSG00000225632	RP5-997D24.3	-2.09	24.82	34.78	41.86	7.34	6.17	7.59	1.31E-28	2.45E-27
ENSG00000232233	RP11-573D15.2	-2.10	4.70	4.31	3.76	1.17	0.99	0.47	7.51E-12	7.43E-11
ENSG00000254682	RP11-660L16.2	-2.10	69.02	66.01	58.58	15.46	13.65	11.76	3.85E-32	7.96E-31
ENSG00000266651	RP11-138I1.3	-2.11	46.32	35.16	46.75	7.76	11.28	7.11	1.06E-15	1.25E-14
ENSG00000242540	AC010729.1	-2.13	2.77	3.15	5.21	1.07	1.01	0.18	6.75E-09	5.61E-08
ENSG00000234614	AL450992.2	-2.14	8.12	13.65	8.51	0.85	2.79	2.14	1.30E-09	1.13E-08
ENSG00000246985	SOCS2-AS1	-2.16	0.67	0.56	0.56	0.17	0.05	0.06	7.87E-07	5.65E-06
ENSG00000267480	RP11-703I16.1	-2.16	2.23	6.86	3.68	0.81	0.00	1.23	1.51E-06	1.06E-05
ENSG00000275632	RP5-967N21.11	-2.16	10.08	9.10	9.86	2.05	1.93	1.24	7.31E-10	6.47E-09
ENSG00000235501	RP4-639F20.1	-2.18	2.32	1.95	1.91	0.42	0.20	0.21	1.06E-06	7.54E-06
ENSG00000215417	MIR17HG	-2.19	8.62	6.12	8.20	1.72	1.39	1.36	4.11E-25	6.86E-24
ENSG00000242125	SNHG3	-2.19	95.25	68.05	80.59	17.47	16.39	14.80	7.73E-119	6.87E-117

ENSG00000259408	RP11-3D4.3	-2.21	0.61	2.05	2.01	0.00	0.63	0.00	8.01E-06	5.28E-05
ENSG00000274667	RP11-31H5.3	-2.22	4.31	2.90	4.12	1.26	0.89	0.00	2.19E-07	1.64E-06
ENSG00000261924	CTD-2561B21.5	-2.22	0.33	0.59	0.92	0.15	0.00	0.15	3.43E-07	2.53E-06
ENSG00000232053	AC009784.3	-2.24	5.48	3.33	3.02	1.00	0.47	0.50	3.91E-10	3.52E-09
ENSG00000233776	LINC01251	-2.24	7.65	7.44	6.20	2.65	0.83	0.44	1.45E-08	1.18E-07
ENSG00000234215	RP5-942I16.1	-2.25	2.83	1.90	1.56	0.31	0.29	0.00	4.35E-06	2.94E-05
ENSG00000235026	DPP10-AS1	-2.27	30.86	31.73	34.41	6.47	5.27	6.30	9.37E-30	1.82E-28
ENSG00000261373	VPS9D1-AS1	-2.28	45.74	43.15	53.68	11.01	7.02	9.00	1.98E-53	6.60E-52
ENSG00000234160	RP11-613M10.6	-2.28	95.41	85.91	105.80	18.55	16.06	17.95	2.66E-27	4.77E-26
ENSG00000227028	SLC8A1-AS1	-2.34	0.14	0.35	0.30	0.05	0.00	0.05	2.52E-07	1.87E-06
ENSG00000273342	KB-1440D3.14	-2.37	8.43	9.69	12.21	1.46	1.37	1.47	2.34E-10	2.14E-09
ENSG00000280018	CH507-154B10.2	-2.37	0.77	0.74	1.81	0.12	0.23	0.00	4.55E-07	3.33E-06
ENSG00000254910	RP11-326C3.7	-2.37	6.98	6.29	4.94	1.09	1.54	0.00	7.73E-08	5.97E-07
ENSG00000235590	GNAS-AS1	-2.38	3.74	3.78	3.24	0.70	0.55	0.53	1.21E-18	1.62E-17
ENSG00000273230	RP11-1246C19.1	-2.41	22.36	31.46	43.34	6.31	4.40	5.50	1.21E-62	4.86E-61
ENSG00000268262	CTC-246B18.8	-2.42	4.01	4.12	2.95	0.73	0.46	0.25	3.54E-09	3.00E-08
ENSG00000277491	RP11-676J12.9	-2.43	3.60	6.05	8.37	0.44	0.41	1.33	1.20E-08	9.84E-08
ENSG00000230649	AC024084.1	-2.43	4.73	6.50	7.10	1.41	1.33	0.00	1.32E-08	1.08E-07
ENSG00000234753	FOXP4-AS1	-2.43	5.44	4.75	6.69	0.46	0.43	2.08	1.16E-11	1.13E-10
ENSG00000266402	SNHG25	-2.48	26.03	20.11	25.56	3.09	2.18	5.44	1.59E-13	1.71E-12
ENSG00000235806	RP4-646N3.1	-2.49	1.82	1.11	1.46	0.18	0.17	0.00	2.30E-07	1.72E-06
ENSG00000272599	RP11-152N13.16	-2.50	12.63	9.76	8.75	2.23	0.63	2.25	5.78E-17	7.24E-16
ENSG00000178248	AP000345.1	-2.51	0.87	1.04	1.36	0.00	0.38	0.00	2.05E-07	1.54E-06
ENSG00000226950	DANCR	-2.51	208.83	212.13	214.10	34.82	34.00	33.67	1.10E-185	1.93E-183
ENSG00000239268	RP11-384F7.2	-2.52	1.77	6.23	6.22	0.39	0.36	0.39	2.22E-08	1.78E-07
ENSG00000260442	ATP2A1-AS1	-2.53	15.77	14.67	13.30	2.21	1.73	2.22	6.18E-16	7.39E-15
ENSG00000259219	CTD-3076O17.2	-2.54	1.06	0.69	1.26	0.09	0.08	0.09	1.62E-08	1.31E-07
ENSG00000259834	RP11-284N8.3	-2.57	3.37	3.26	4.21	0.25	0.70	0.74	3.90E-20	5.52E-19
ENSG00000255571	MIR9-3HG	-2.57	4.65	3.76	4.36	0.79	0.44	0.73	1.14E-34	2.51E-33
ENSG00000268592	RAET1E-AS1	-2.58	1.62	1.63	3.20	0.35	0.33	0.00	7.12E-09	5.91E-08
ENSG00000235033	RP11-61I13.3	-2.63	1.85	1.99	2.54	0.31	0.35	0.22	1.32E-22	2.04E-21
ENSG00000236856	AC105393.1	-2.64	4.63	3.29	2.75	0.00	0.37	0.39	2.77E-08	2.21E-07
ENSG00000276292	RP11-33N14.5	-2.67	10.10	9.26	13.14	0.00	1.89	1.01	5.21E-09	4.36E-08
ENSG00000233589	RP4-694A7.2	-2.69	2.85	3.25	3.36	0.22	0.42	0.23	3.24E-10	2.94E-09
ENSG00000272798	CTA-390C10.9	-2.70	3.24	1.91	2.50	0.00	0.33	0.00	7.21E-08	5.58E-07
ENSG00000225218	AP001628.6	-2.70	9.61	2.88	5.29	0.00	0.00	0.76	6.59E-08	5.11E-07
ENSG00000261786	RP4-555D20.2	-2.80	0.50	0.49	0.51	0.00	0.04	0.09	5.35E-10	4.79E-09
ENSG00000229418	RP11-35J23.1	-2.84	1.52	0.77	0.56	0.00	0.10	0.00	9.46E-09	7.79E-08
ENSG00000196758	AC079612.1	-2.85	0.79	0.93	0.47	0.06	0.11	0.00	2.52E-10	2.30E-09
ENSG00000259287	RP11-3D4.2	-2.87	6.26	4.39	5.36	0.38	0.00	1.15	5.23E-11	4.94E-10
ENSG00000243479	MNX1-AS1	-2.89	23.48	24.17	23.37	3.30	1.94	3.32	3.86E-35	8.64E-34
ENSG00000230526	RP11-472G21.2	-2.94	63.70	82.37	75.47	7.76	9.61	8.23	4.64E-47	1.35E-45
ENSG00000235688	AC116614.1	-2.94	15.96	18.00	18.78	1.59	2.25	1.60	1.56E-20	2.24E-19
ENSG00000226806	AC011893.3	-2.98	36.97	36.65	41.17	5.07	4.12	3.95	3.22E-70	1.45E-68
ENSG00000176912	TYMSOS	-2.98	9.62	10.78	13.68	0.88	1.24	1.33	1.08E-22	1.67E-21
ENSG00000249084	RP11-376O6.2	-3.01	2.08	2.84	4.58	0.00	0.00	0.57	1.89E-10	1.73E-09
ENSG00000272512	RP11-54O7.17	-3.06	13.97	9.62	14.66	1.85	0.87	1.35	3.31E-39	8.18E-38
ENSG00000283638	MIR92A2	-3.15	1.37	0.89	1.34	0.00	0.21	0.11	3.64E-15	4.23E-14
ENSG00000229191	RP11-168O16.1	-3.18	2.84	2.58	2.15	0.06	0.17	0.62	1.73E-21	2.58E-20
ENSG00000260947	RP11-384P7.7	-3.20	1.39	2.42	2.08	0.18	0.23	0.06	6.70E-19	9.06E-18
ENSG00000268798	CTB-25B13.5	-3.23	9.96	12.11	14.09	2.14	1.10	0.39	1.10E-27	2.00E-26
ENSG00000257060	RP11-266O8.1	-3.31	0.32	0.67	0.59	0.00	0.00	0.00	5.27E-11	4.98E-10
ENSG00000260302	RP11-973H7.1	-3.39	21.97	20.15	24.53	1.85	2.06	1.53	1.45E-46	4.18E-45
ENSG00000229941	AC012499.1	-3.57	1.58	3.77	4.65	0.00	0.00	0.00	4.87E-13	5.12E-12
ENSG00000230830	ARPP21-AS1	-3.58	10.10	5.76	6.36	0.40	0.00	0.40	1.99E-16	2.44E-15

ENSG00000232487	RASA3-IT1	-3.70	44.26	47.16	45.33	1.13	3.72	4.55	2.56E-38	6.22E-37
ENSG00000267665	RP11-13K12.2	-3.71	1.23	1.39	1.54	0.09	0.04	0.05	7.00E-22	1.06E-20
ENSG00000228203	RNF144A-AS1	-3.72	1.14	2.02	2.41	0.14	0.09	0.05	3.12E-25	5.23E-24
ENSG00000245248	USP2-AS1	-3.81	2.01	2.49	1.90	0.36	0.04	0.05	9.23E-29	1.74E-27
ENSG00000236117	RP4-754E20_A.5	-3.83	3.91	5.19	5.89	0.23	0.00	0.23	1.96E-19	2.71E-18
ENSG00000260123	RP11-326A19.4	-3.86	21.95	24.51	21.64	1.44	1.35	0.72	6.10E-35	1.36E-33
ENSG00000258748	CTD-2223O18.1	-3.88	8.04	13.88	15.38	0.93	0.44	0.00	2.10E-21	3.12E-20
ENSG00000256008	RP11-728G15.1	-4.14	12.06	11.90	14.24	0.19	0.72	0.77	1.08E-38	2.65E-37
ENSG00000225518	RP11-396C23.2	-4.21	5.96	6.61	4.72	0.00	0.25	0.00	1.23E-21	1.84E-20
ENSG00000236656	RP11-144L1.4	-4.23	1.13	1.46	1.74	0.00	0.00	0.00	1.70E-19	2.35E-18
ENSG00000227459	AC079612.2	-4.65	24.16	33.07	26.61	1.23	0.58	0.00	5.56E-35	1.24E-33
ENSG00000272789	RP11-286H15.1	-4.72	91.58	78.16	81.63	4.78	1.42	2.79	4.43E-110	3.54E-108
ENSG00000259200	RP11-718O11.1	-5.00	8.32	9.58	11.36	0.17	0.32	0.00	6.54E-43	1.75E-41
ENSG00000274840	RP11-320G10.1	-5.04	15.58	24.25	19.69	0.00	0.00	0.00	5.34E-31	1.07E-29

Table S2. List of lncRNAs regulated in the same direction both in Jurkat cells after PMA/ionomycin treatment and in FL samples.

			Jurkat			FL	
**Geneid**	**Gene name**	**logFC**	**NS**	**SW4DR**	**logFC**	**FL**	**GCB**
ENSG00000204054	LINC00963	7.45	0.05	10.59	5.03	1.10	0.03
ENSG00000233093	LINC00892	6.55	3.47	302.96	7.39	2.72	0.01
ENSG00000178977	LINC00324	4.37	1.46	28.90	1.04	2.97	1.27
ENSG00000274536	MIR223HG	4.20	13.39	219.45	3.69	8.35	0.43
ENSG00000267414	RP11-456K23.1	3.12	0.12	1.71	0.56	1.54	0.91
ENSG00000232533	AC093673.5	2.69	10.44	62.61	1.42	4.41	1.45
ENSG00000260231	JHDM1D-AS1	2.52	1.02	5.52	1.81	1.98	0.48
ENSG00000245532	NEAT1	2.49	19.00	98.22	1.10	17.58	7.01
ENSG00000246898	LINC00920	2.24	2.58	11.41	6.27	0.75	0.01
ENSG00000234883	MIR155HG	2.13	1.11	4.97	1.61	5.25	1.46
ENSG00000271151	RP11-394I13.2	2.05	0.78	3.49	0.88	0.76	0.36
ENSG00000261366	MANEA-AS1	1.90	0.76	2.90	1.14	1.08	0.42
ENSG00000206028	CTA-373H7.7	1.88	0.33	1.29	2.43	11.25	1.63
ENSG00000256576	RP13-977J11.2	1.69	17.79	53.05	3.82	1.93	0.11
ENSG00000266947	RP11-799D4.4	1.68	2.85	8.43	2.54	0.45	0.07
ENSG00000223891	OSER1-AS1	1.60	1.32	3.86	0.56	0.55	0.33
ENSG00000272144	CTD-2035E11.5	1.59	1.86	5.51	1.27	0.93	0.33
ENSG00000267838	AC008746.12	1.53	1.61	4.71	1.03	0.80	0.33
ENSG00000271803	RP1-63M2.5	-1.53	26.41	8.18	-3.49	0.11	1.47
ENSG00000273162	RP11-108L7.15	-1.67	13.16	3.92	-1.55	0.46	1.23
ENSG00000261431	RP4-616B8.4	-1.68	5.79	1.13	-1.08	0.29	0.57
ENSG00000228649	AC005682.5	-1.93	9.98	2.35	-0.62	0.58	0.80
ENSG00000255717	SNHG1	-2.02	203.68	46.27	-1.20	0.67	1.41
ENSG00000266651	RP11-138I1.3	-2.11	42.74	8.72	-1.22	0.68	1.41
ENSG00000215417	MIR17HG	-2.19	7.65	1.49	-1.66	0.27	0.84
ENSG00000273230	RP11-1246C19.1	-2.41	32.39	5.40	-0.55	0.62	0.80
ENSG00000261786	RP4-555D20.2	-2.80	0.50	0.04	-1.46	0.18	0.52

Table S3. List of the genes with the strongest positive or negative expression correlation (pearson) with LINC00892 in all cell types analyzed by Monaco et al. (Monaco et al. Cell Reports). Genes marked with an asterisk are known to be high expressed in CD4+ Follicular T helper cells.

Gene	Pearson	p-value
LINC00892	1	0
CCR4*	0.858	3.50E-34
CD40LG*	0.858	3.73E-34
ZDHHC11B	0.833	1.66E-30
ICOS*	0.832	2.15E-30
CLIC5	0.827	9.79E-30
PDGFB	0.811	7.79E-28
PDCD1*	0.809	1.35E-27
INPP4B	0.808	1.73E-27
CCR8	0.796	3.57E-26
EMBP1	0.791	1.10E-25
ZNF831	0.790	1.53E-25
RNF214	0.787	2.89E-25
FBXL8	0.786	3.86E-25
CLDND1	0.784	6.35E-25
TNFRSF25	0.784	6.53E-25
RPL5P13	0.783	7.89E-25
IL7R	0.782	9.80E-25
CDC14A	0.781	1.10E-24
UBASH3A	0.780	1.42E-24
CYBB	-0.684	4.66E-17
NCF1B	-0.685	4.12E-17
RAB31	-0.686	3.93E-17
PIK3AP1	-0.689	2.44E-17
RHOQ	-0.690	1.99E-17
CORO1C	-0.692	1.62E-17
P2RX1	-0.692	1.62E-17
CBFA2T3	-0.693	1.37E-17
PECAM1	-0.693	1.26E-17
MEF2C	-0.694	1.18E-17
NCF1C	-0.694	1.15E-17
PLCG2	-0.696	7.75E-18
CD68	-0.697	6.70E-18
GAPT	-0.701	3.83E-18
HHEX	-0.703	2.99E-18
METTL7A	-0.709	1.12E-18
BTK	-0.715	3.97E-19
FES	-0.723	1.12E-19
LYN	-0.739	6.14E-21
SYK	-0.747	1.45E-21

Table S4. List of oligonucleotides used.

Gene	Forward Primer (5' - 3')	Reverse Primer (5' - 3')
FASL	TCTACCAGCCAGATGCACAC	CAGAGGCATGGACCTTGAGT
BCL6	TGAGAAGCCCTATCCCTGTG	CTGGCTTTTGTGACGGAAAT
DUSP10	GCGGCAGTACTTTGAAGAGG	GTCATGGTCATCCGAGTGTG
LINC00892	CTACTGGGCTGGAAGTCAGG	CCTCAGGTTCTCCAGTCAGC
PAX5	QuantiTect Primer Assay QT00021399 (Qiagen)	
CDKN1A	GACTCTCAGGGTCGAAAACG	GGCGTTTGGAGTGGTAGAAA
IL2	AACTCACCAGGATGCTCACA	GCACTTCCTCCAGAGGTTTG
CD40LG	QuantiTect Primer Assay QT00000343 (Qiagen)	
G6PD	CGTCACCAAGAACATTCACG	AGGAGATGTGGTTGGACAGC
GUSB	TTTGCCGATTTCATGACTGA	CCTGGTTTCATTGGCAATCT
MALAT1	TGGGGGAGTTTCGTACTGAG	TTTCTCCAGGACTTGGCAGT
